# Ser/Thr protein kinase PrkC-mediated regulation of GroEL is critical for biofilm formation in *Bacillus anthracis*

**DOI:** 10.1038/s41522-017-0015-4

**Published:** 2017-03-07

**Authors:** Gunjan Arora, Andaleeb Sajid, Richa Virmani, Anshika Singhal, C. M. Santosh Kumar, Neha Dhasmana, Tanya Khanna, Abhijit Maji, Richa Misra, Virginie Molle, Dörte Becher, Ulf Gerth, Shekhar C. Mande, Yogendra Singh

**Affiliations:** 1 CSIR-Institute of Genomics and Integrative Biology, Delhi, 110007 India; 2 National Centre for Cell Science, NCCS Complex, University of Pune Campus, Ganeshkhind, Pune , 411007 Maharashtra India; 3 DIMNP, CNRS, University of Montpellier, Montpellier, France; 4 Institute of Microbiology, Ernst-Moritz-Arndt-University Greifswald, Greifswald , D-17487 Germany; 5 Department of Zoology, University of Delhi, Delhi , 110007 India

## Abstract

PrkC is a conserved Ser/Thr protein kinase encoded in *Bacillus anthracis* genome. PrkC is shown to be important for *B. anthracis* pathogenesis, but little is known about its other functions and phosphorylated substrates. Systemic analyses indicate the compelling role of PrkC in phosphorylating multiple substrates, including the essential chaperone GroEL. Through mass spectrometry, we identified that PrkC phosphorylates GroEL on six threonine residues that are distributed in three canonical regions. Phosphorylation facilitates the oligomerization of GroEL to the physiologically active tetradecameric state and increases its affinity toward the co-chaperone GroES. Deletion of *prkC* in *B. anthracis* abrogates its ability to form biofilm. Overexpression of native GroEL recovers the biofilm-forming ability of *prkC* deletion strain. Similar overexpression of GroEL phosphorylation site mutants (Thr to Ala) does not augment biofilm formation. Further analyses indicate the phosphorylation of GroEL in diverse bacterial species. Thus, our results suggest that PrkC regulates biofilm formation by modulating the GroEL activity in a phosphorylation-dependent manner. The study deciphers the molecular signaling events that are important for biofilm formation in *B. anthracis*.

## Anthrax bacteria: a step in the pathway to biofilms

An enzyme that adds phosphate groups to other proteins, PrkC, mediates molecular signaling events that allow anthrax bacteria to form biofilms. *Bacillus anthracis* is widely used as a model to explore the formation of biofilms that allows many bacterial infections to resist immune defenses. An international research team led by Yogendra Singh and Andaleeb Sajid at the CSIR-Institute of Genomics and Integrative Biology in Delhi, India, studied the bacterial protein kinase PrkC. The researchers found that PrkC phosphorylates a “chaperone” protein that assist the assembly and disassembly of other protein-based structures. This signaling protein and the chaperone help in biofilm formation. Establishing this link in the signaling chain leading to biofilms will guide future research to combat the role of biofilms in disease.

## Introduction

*Bacillus anthracis* (*B. anthracis*) is a bacterial pathogen that causes anthrax and has been historically used as a model organism to understand the bacterial response during infection.^1^ The lifecycle of *B. anthracis* comprises of vegetative and sporulation phases and possess the ability to form capsules and biofilms.^2, 3^ *B. anthracis*, *Bacillus cereus*, and *Bacillus thuringiensis* are the three major species comprising the pathogenic *Bacillus cereus* group. These bacteria are difficult to eradicate, both in the environment and during infection, mainly due to the efficient development of spores and biofilms. *B. anthracis* cells readily form biofilms under stagnant conditions in environment and protect the vegetative cells, which continue to divide within biofilm communities.^4^ The cells can eventually sporulate and disseminate, thus causing exponential increase in bacterial cell number under favorable conditions. During infection, pathogens tend to sporulate or form biofilms on epithelial cells, enabling the bacteria to escape innate immune responses and become antibiotic-resistant.^5–8^ Thus, entering the sporulation phase or forming biofilm is a survival strategy for bacteria.^3, 9–11^ The mechanism of biofilm formation remains poorly understood in *B. anthracis*, although biofilm-forming cells are suggested to be particularly resistant to high levels of antibiotic treatments.^3^ Further, *Bacillus* forms communities that have both biofilm and spores, and the biofilm disassembly leads to spore release. It is plausible that for planktonic cells biofilm-associated growth provides fitness advantage.

Bacterial cells sense extracellular signals and respond in a concerted manner during morphogenesis. The role of molecular signaling events occurring inside *B. anthracis* remains unknown. The cascades initiate at cellular surface and culminate in the modulation of defined set of proteins, leading to specific cellular response, such as biofilm formation.^12^ In *B. anthracis*, PrkC is the only known Ser/Thr protein kinase (STPK) with a sensor domain capable of receiving external signals.^13–16^ PrkC senses muropeptides through the extracellular C-terminal PASTA domains,^17–20^ but its downstream signaling events are not yet known. Homologs of PrkC are conserved in many bacteria such as *Mycobacterium*, *Streptomyces*, *Staphylococcus*, *Corynebacterium*, and other diverse gram-positive bacteria along with its cognate Ser/Thr phosphatase (PrpC), and is known to regulate vital functions.^16, 19, 21–28^ PrkC plays an important role in virulence of *B. anthracis* and is important for survival in macrophages during infection.^15, 16^ PrkC is capable of transducing signals efficiently during environmental stress conditions and has been proposed to function even in the spores.^14, 17^

In view of these facts, we aimed to explore the importance of *B. anthracis* PrkC in the cellular signaling events. To identify its role(s) in vivo, we searched for proteins phosphorylated by PrkC. Further, using a *B. anthracis* deletion strain of *prkC* (*Bas*Δ*prkC*), we investigated its role in regulation of biofilm formation. In accordance to our hypothesis, deletion of *prkC* resulted in complete loss of biofilm formation and possibly altered cell-to-cell adherence properties. Our results indicated that PrkC may regulate biofilm formation by activating an essential chaperone GroEL. Thus, this study describes the novel signaling pathway involving the conserved STPK and chaperone in *B. anthracis* biofilm development.

## Results

### PrkC regulates *B. anthracis* biofilm formation

**Fig. 1:**
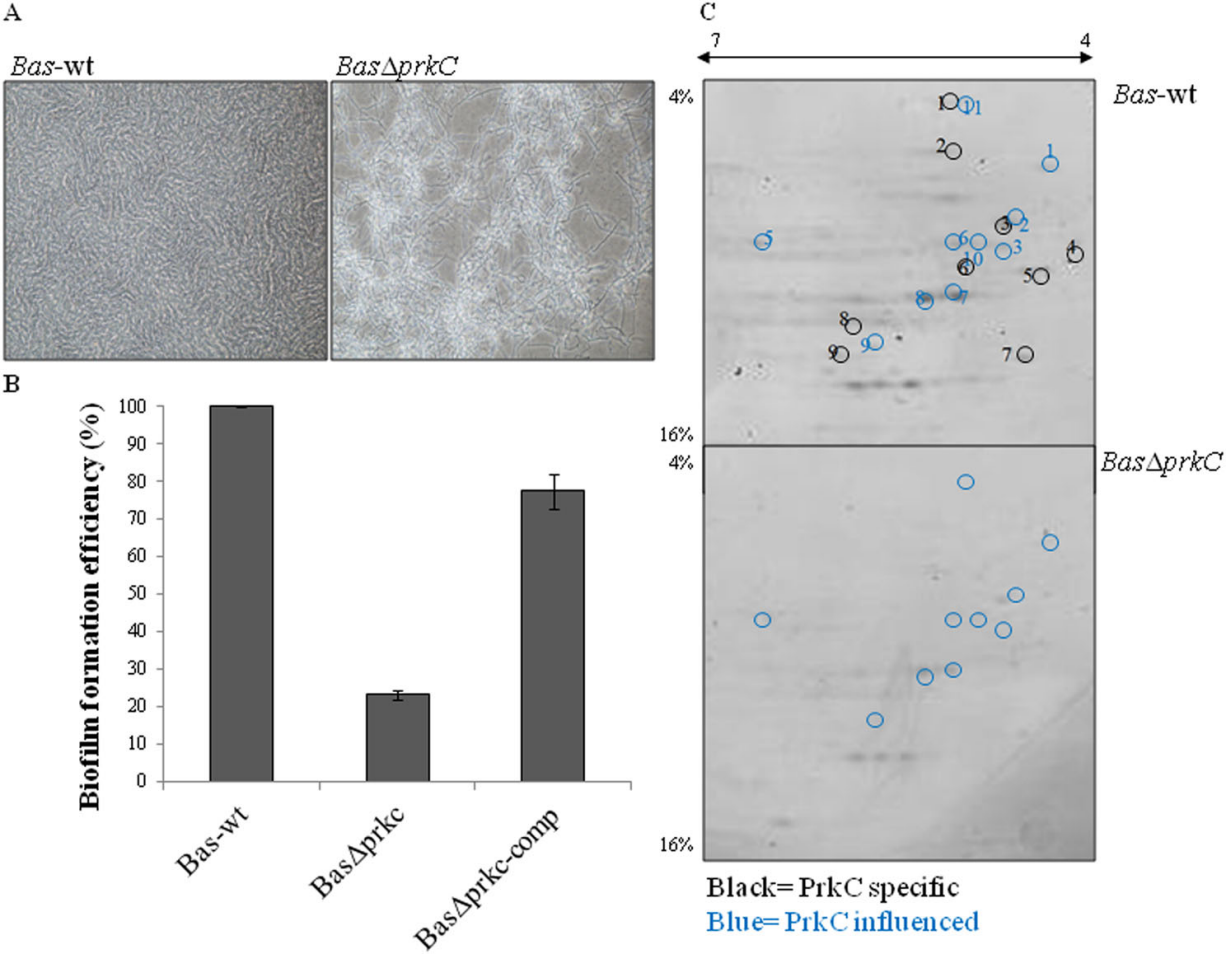
PrkC-dependent biofilm formation in *B. anthracis* and identification of in vivo substrates: **a** Biofilm formation in 3-day static culture of *Bas*-wt and *Bas*Δ*prkC* strains after staining with crystal violet as observed under microscope. **b** Efficiency of biofilm formation in *Bas*-wt, *Bas*Δ*prkC*, and complemented *Bas*Δ*prkC* strains were calculated by crystal violet assay. Extent of biofilm formation was calculated considering *Bas*-wt as 100%. The experiment was performed thrice and the error bars indicate the standard error (SE) of three independent values. **c** *Bas*-wt and *Bas*Δ*prkC* lysates were separated by two-dimensional PAGE using 4–16% gradient gels and visualized with Pro-Q diamond phospho-specific staining. These proteins were subsequently analyzed by staining the gels with SyproRuby stain (Fig. S1). The more intense (*blue* numbered, PrkC-influenced) or unique spots (*black* numbered, PrkC-specific) in *Bas*-wt as compared with *Bas*Δ*prkC* were analyzed by mass spectrometry (Table 1). The bands are also encircled with corresponding color

To understand the effect of PrkC in *B. anthracis* biofilm formation, we utilized the deletion strain *Bas*Δ*prkC*,^17^ and compared it with the wild-type *Bas*-wt. We observed that deletion of *prkC* did not cause any change in bacterial culture growth rate, as described earlier.^15, 16^ However, *Bas*Δ*prkC* cells appeared to have loose settling and surface-adherence property as compared with *Bas*-wt. Adherence to the growth surface is achieved by forming biofilms at the liquid–air interface. To test the biofilm formation, *Bas*-wt and *Bas*Δ*prkC* were cultured under static conditions. We observed that *Bas*-wt cells were able to form biofilm within 3 days, while this property was lost in *Bas*Δ*prkC* (Fig. 1a, b). To further substantiate this observation, deletion strain was complemented with *prkC* (*Bas*Δ*prkC*-comp). Biofilm-forming ability of all three strains, *Bas*-wt, *Bas*Δ*prkC*, and *Bas*Δ*prkC*-comp, was compared using the quantitative crystal violet assay. *Bas*Δ*prkC*-comp regained the property of biofilm formation to a level similar to *Bas*-wt (Fig. 1b), indicating that PrkC is involved in biofilm formation. Being a regulatory protein kinase, PrkC exerts its effects through signal transduction pathways involving phosphorylation of its substrates. Thus, changes in biofilm formation may be attributed to PrkC-mediated phosphorylation of specific substrate(s), as discussed in the following sections.

### Identification of PrkC substrates

To identify the targets of PrkC *in B. anthracis*, the lysates of *Bas*-wt and *Bas*Δ*prkC* were resolved by two-dimensional gradient SDS-PAGE. The gels were stained with phosphorylation-specific Pro-Q Diamond stain and the signal was normalized to total protein levels by SYPRO Ruby staining. We selected two types of protein spots in Pro-Q stained gels (Fig. 1c and S1): (1) proteins that are present in both the strains but have higher intensity in *Bas*-wt as compared with *Bas*Δ*prkC* (PrkC-influenced), and (2) proteins in *Bas*-wt that are absent in *Bas*Δ*prkC* (PrkC-specific). These differentially phosphorylated protein spots were identified by mass spectrometry (Table 1). We could identify 20 different proteins, of which 9 were PrkC-specific while 10 were PrkC-influenced. Our analysis identified GroEL phosphorylation as a signature of PrkC activity in *B. anthracis*.

**Table 1:** Differentially phosphorylated proteins identified by mass spectrometry

Spot number	Gene ID	Protein encoded
PrkC-specific		
1	Bas0107, *fusA*	Elongation factor-G
2	Bas3470	Transketolase
3	Bas0253, *groEL*	GroEL, 60 kDa chaperone
4	Bas4937, *murB*	UDP-N-acetylenolpyruvoylglucosamine reductase
5	Bas4985, *eno*	Phosphopyruvate hydratase
6	Bas5155	ATP synthase F0F1 subunit beta
7	Bas3882	Pyruvate dehydrogenase E1 component subunit beta
8	Bas4070	Leucine dehydrogenase
9	Bas3677, *tsf*	Elongation factor-Ts
PrkC-influenced		
1	Bas4213, *dnaK*	Molecular chaperone DnaK
2	Bas0253, *groEL*	GroEL, 60 kDa chaperone
3	Bas4792, *pepA*	Leucyl aminopeptidase
4	Bas0330	Alkyl hydroperoxide reductase
5	Bas0295	1-Pyrroline-5-carboxylate dehydrogenase
6	Bas3392	DNA topoisomerase IV subunit B, DNA gyrase
7, 8	Bas0108, *tuf*	Elongation factor-Tu
9	Bas4297, *aspS*	Aspartyl-tRNA synthetase
10	Bas1408, *rpsA*	30S ribosomal protein S1
11	Bas3408	Aconitate hydratase

*Note*: The proteins that showed higher intensity were grouped as “PrkC-influenced”, denoting increased phosphorylation in presence of PrkC. The proteins that were newly phosphorylated were grouped under “PrkC-specific”, denoting the PrkC-specific phosphorylated proteins (Fig. 1c, S1)

To confirm the substrates of PrkC, we followed two more strategies: immunoprecipitation and phosphoenrichment. Immunoprecipitation was performed with antibodies specific to pSer and pThr using *B*. *anthracis* whole cell protein lysate. Using this approach, seven prominently phosphorylated proteins were identified by mass spectrometry (Table 2). Phosphoenrichment was performed using lysates of strains *Bas*-wt and *Bas*Δ*prkC* (using Qiagen Phosphoenrichment kit). Equal amounts of phospho-enriched proteins from both the samples were analyzed by SDS-PAGE to detect the differentially enriched proteins because of variable phosphorylation, followed by mass spectrometry to identify the PrkC phosphorylated proteins (Table 3).

**Table 2:** List of proteins identified by immunoprecipitation with *α*-pSer/*α*-pThr antibodies

Protein identified	Gene ID	MS score	Sequence coverage
Elongation factor Tu	Bas0108	99	50.5%
Superoxide dismutase	Bas5300	105	60.3%
GroEL, 60 kDa chaperone	Bas0253	97	44.1 %
Aldehyde dehydrogenase	Bas3348	113	54.5%
1-Pyrroline-5-carboxylate dehydrogenase	Bas0295	197	52.0%
Formate acetyltransferase	Bas0481	94	42.3%
Alcohol dehydrogenase	Bas2111	144	59.7%

**Table 3:** Phospho-enriched proteins in *Bas*-wt identified by mass spectrometry

Protein identified	Gene ID	MS score	Sequence coverage
Leucine dehydrogenase	Bas4070	151	32%
GroEL, 60 kDa chaperone	Bas0253	239	45%
Short-chain Enoyl-coA hydratase	Bas4420	86	33%
Nucleoside diphosphate kinase	Bas1425	150	53%
Respiratory nitrate reductase, alpha subunit	Bas1977	160	33%
Phosphoglucomutase/Phosphomannomutase family protein	Bas4790	213	58%
Zinc-containing alcohol dehydrogenase	Bas0641	118	34%
Hypoxanthine-guanine phosphoribosyltransferase	Bas0063	85	45%
Hypothetical protein, HTH arsenical resistance operon repressor domain	Bas4146	125	50%

**Fig. 2:**
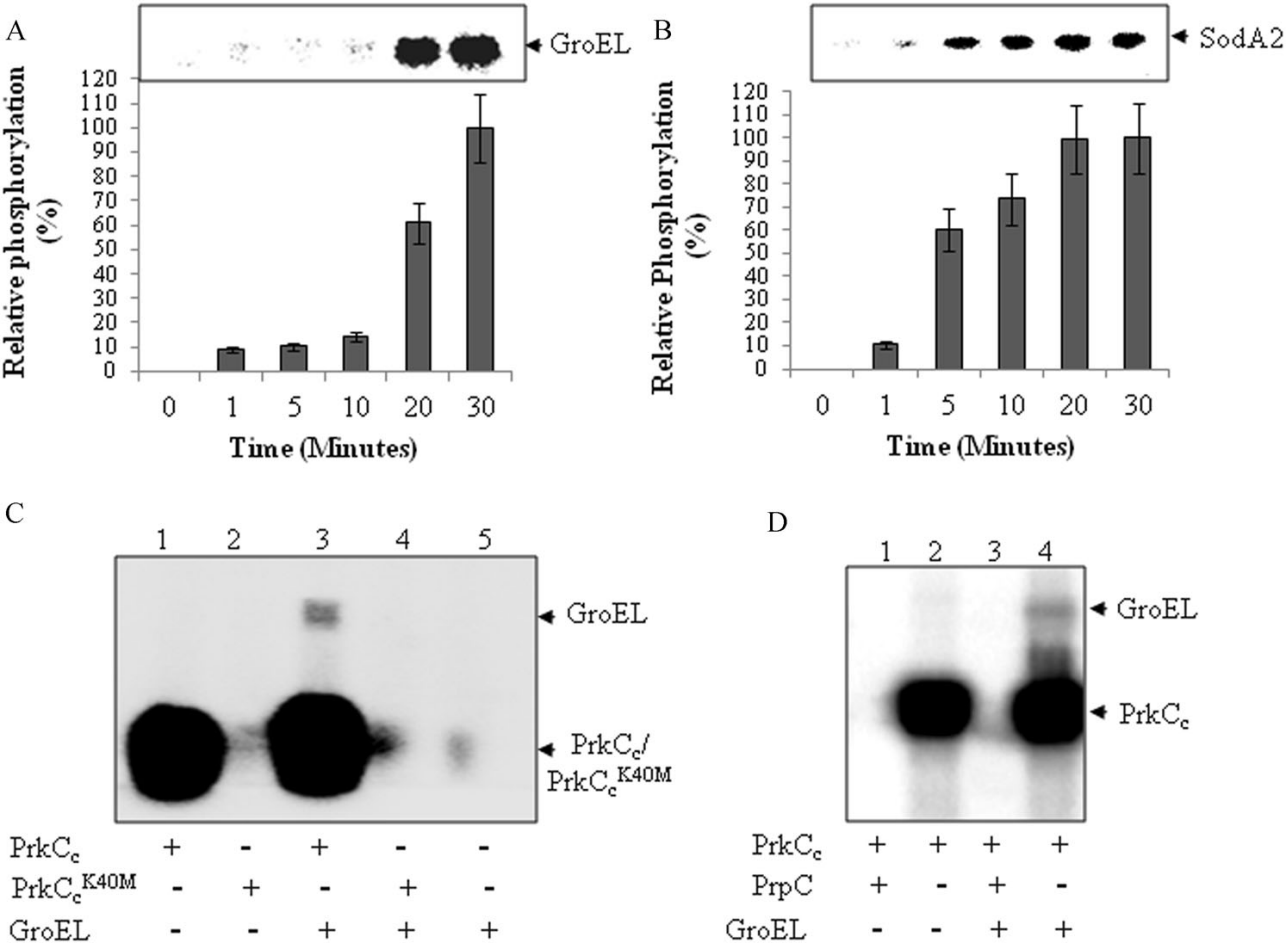
Time-dependent phosphorylation kinetics of PrkC and reversible phosphorylation of GroEL: To validate GroEL as PrkC substrate (Tables 1, 2, and 3), we used recombinant GroEL (57.5 kDa) for in vitro kinase assays using the kinase domain of PrkC [PrkC_c_, 1-337 aa, ~40 kDa^14^]. Autoradiogram shows Time-dependent phosphorylation of GroEL (**a**) and SodA2 (**b**), with autophosphorylated kinase (using cold ATP). Phosphorylation intensity at 30 min time point was taken as 100% and relative phosphorylation was calculated followed by normalization with the protein amount (Fig. S2). The corresponding autoradiogram is shown above the bar graph. The assay was performed twice and intensities were calculated three times each. Error bars represent SE of six independent calculations. **c** Autoradiogram showing in vitro phosphorylation of GroEL. PrkC catalytic domain (PrkC_c_, 1 µg) was used for phosphorylation (for 30 min) of purified GroEL (5 µg) and the kinase dead mutant (PrkC_c_-K40M) was used as a negative control. No phosphorylation was observed in the control reactions when the kinase inactive mutant PrkC_c_-K40M was used.^14^ **d** Autoradiogram showing in vitro dephosphorylation of PrkC-phosphorylated GroEL by Ser/Thr phosphatase PrpC (1 µg)

Three substrates identified in screening were independently validated by in vitro phosphorylation assay using recombinant PrkC. Time-dependent phosphorylation kinetics showed optimal time for efficient in vitro phosphorylation (Fig. 2a, b, S2). Ef-Tu that has previously been shown to be phosphorylated by PrkC (and homologs) in *B. anthracis*, *Bacillus subtilis* (*B. subtilis*), and *M. tuberculosis* was used as a positive control to corroborate our results.^14, 20, 21, 29^ The experiment confirmed phosphorylation of SodA2, Ef-G, and GroEL by PrkC (Fig. 2, S3). PrkC-K40M mutant was used as negative control, which is incapable of carrying out phosphorylation.^14, 15^ Subsequently, to test the reversibility of phosphorylation, phosphorylated GroEL was incubated with PrpC, which led to its dephosphorylation (Fig. 2d). These results demonstrate that the kinase-phosphatase pair PrkC-PrpC regulates GroEL phosphorylation reversibly. Since the chaperone protein GroEL is involved in biofilm formation in *Streptococci* and *Mycobacteria*,^30, 31^ and was the only phosphorylated protein identified by all three methods, we investigated the role of PrkC in regulating *B. anthracis* GroEL.

### Expression and phosphorylation status of GroEL in *B. anthracis*

**Fig. 3:**
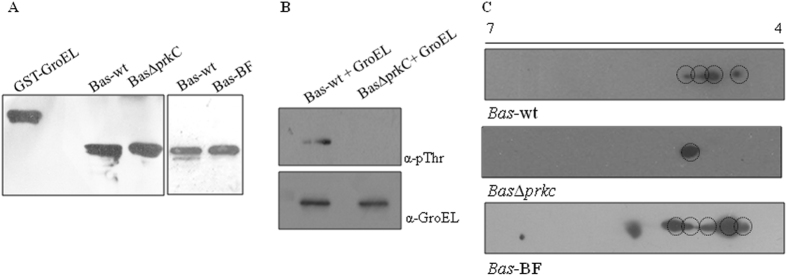
GroEL expression and phosphorylation in *B. anthracis*: **a** Immunoblots show the expression of GroEL in *B. anthracis* cell lysates. Equal amounts of lysates (5 µg each) were used and probed with anti-GroEL antibodies. Purified GST-GroEL (0.5 µg) was taken as the positive control. *Left panel* shows the comparative expression of GroEL in *Bas*-wt and *Bas*Δ*prkC*. *Right panel* shows the comparative expression of GroEL in *Bas*-wt and *Bas*BF. **b** *groEL* was overexpressed and purified from *B. anthracis* strain, followed by immunoblotting with anti-pThr antibody. GroEL was phosphorylated in *Bas-wt* whereas no phosphorylation was observed with GroEL purified from *BasΔprkC*. **c** Two-dimensionally separated cell lysates of *B. anthracis* were probed with anti-GroEL antibodies to assess the stoichiometry of phosphorylation of native GroEL. Multiple species were observed in *Bas*-wt as compared with *Bas*Δ*prkC*, showing the in vivo phosphorylation of GroEL specifically by PrkC. GroEL was hyper-phosphorylated during biofilm formation (*Bas*BF) as observed by the increased number of spots (*third panel*)

To understand the role of GroEL in biofilm formation, we compared its expression profile in *Bas*-wt and *Bas*Δ*prkC* cells (grown in liquid media with shaking) with *Bas*-wt biofilms (grown in static condition), using immunoblotting with anti-GroEL antibodies. There was no change in expression of GroEL in the *Bas*Δ*prkC* strain as compared with *Bas*-wt, but the expression increased marginally in biofilm-forming cells, indicating that it might be important under such conditions (Fig. 3a). To understand the in vivo status of GroEL phosphorylation, we overexpressed GroEL in *Bas*-wt and *Bas*Δ*prkC*. Overexpressed GroEL purified from *Bas*-wt and *Bas*Δ*prkC* was subjected to immunoblotting using anti-pThr antibodies and the result shows PrkC-mediated phosphorylation of GroEL in *Bas*-wt (Fig. 3b). Further to understand the stoichiometry of GroEL phosphorylation, we parsed the phosphorylated and unphosphorylated isoforms. Whole cell protein extracts from *Bas*-wt and *Bas*Δ*prkC* strains were resolved by two-dimensional PAGE followed by immunoblotting using anti-GroEL antibody. In *Bas*Δ*prkC*, we observed only one GroEL protein isoform that migrated to an approximate pI of 4.7 (Fig. 3c). However in *Bas*-wt cells, we identified four isoforms of GroEL, of which one was at pI similar to that in *Bas*Δ*prkC* strain (i.e., ~4.7), and the other spots migrated to a lower pI range nearing 4.0 (Fig. 3c). Since the phosphorylated species are more acidic in nature, we concluded that the additional GroEL isoforms refer to the phosphorylated species. This indicated the presence of phosphorylated species of GroEL in *Bas*-wt as compared with single species (unphosphorylated) in *Bas*Δ*prkC* strain. The stoichiometry of GroEL phosphorylation was subsequently assessed in biofilm-forming cells (*Bas*-BF). We identified multiple isoforms with lower pI as compared with *Bas*Δ*prkC* strain and *Bas*-wt cells (Fig. 3c). These results clearly indicate that GroEL expression and its phosphorylation are associated with biofilm formation, and it is imperative to analyze the underlying regulatory process.

### Co-expression of GroEL with PrkC/PrpC and effect of phosphorylation on oligomerization

**Fig. 4:**
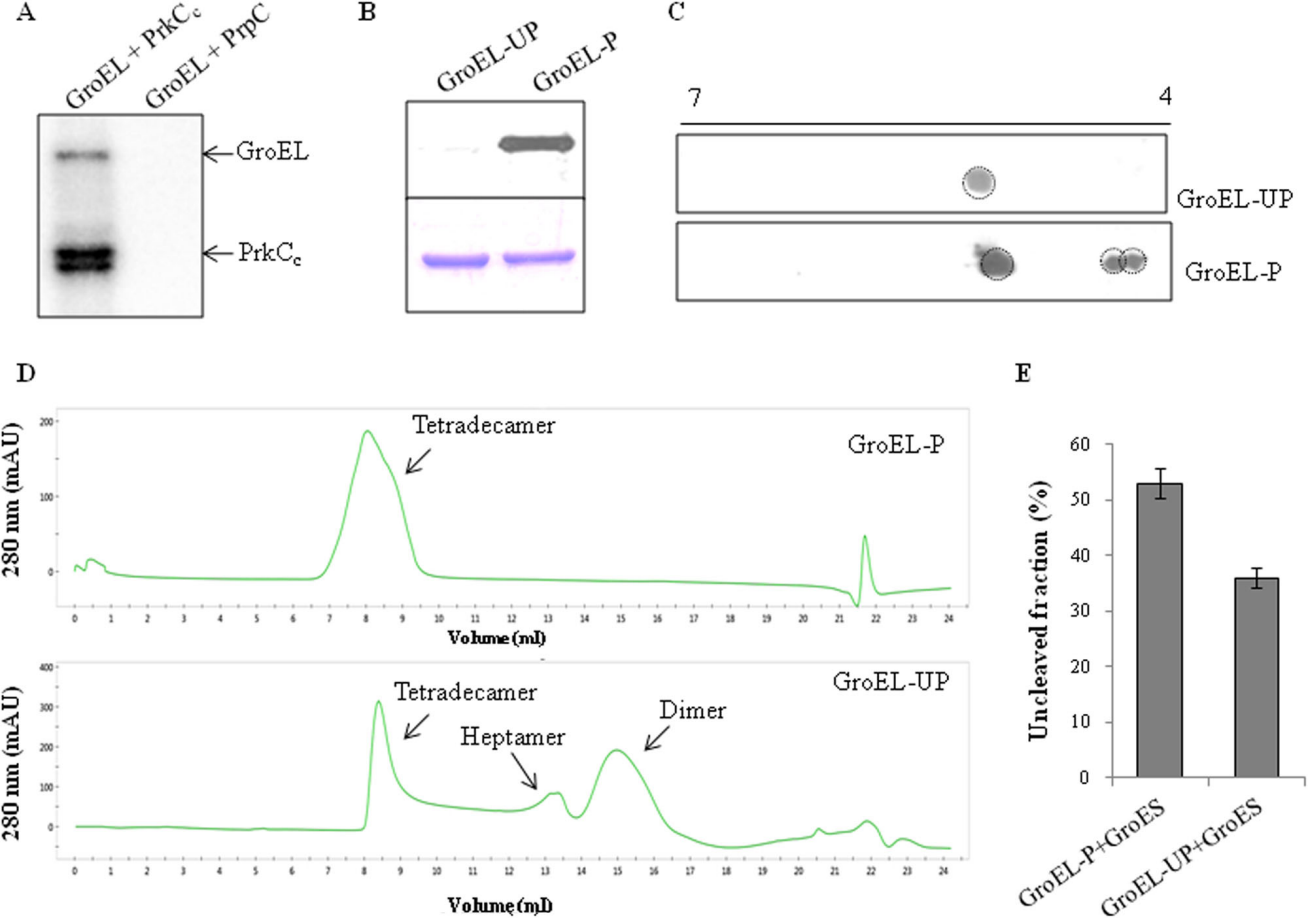
Co-expression of GroEL with PrkC and PrpC in *E. coli* and effect of phosphorylation on GroEL: **a** *E. coli* BL21 cells overexpressing His_6_-GroEL with either PrkC_c_ or PrpC were metabolically labeled using [^32^P]orthophosphoric acid. As shown in the autoradiogram, GroEL is phosphorylated in the presence of PrkC. Due to its affinity for GroEL, PrkC_c_ was also co-precipitated. **b** Purified His_6_-GroEL co-expressed with either PrkC_c_ or PrpC were resolved by SDS-PAGE. The gel was stained with Pro-Q stain and analyzed by Typhoon imager. GroEL was phosphorylated when co-expressed with PrkC_c_ (GroEL-P), while co-expression with PrpC does not result in GroEL phosphorylation (GroEL-UP). **c** To understand the stoichiometry of phosphorylation of GroEL-P and GroEL-UP, the purified proteins (1 µg each) were separated by two-dimensional PAGE (pI range 4–7). The gels were immunoblotted on nitrocellulose membrane and developed using anti-GroEL antibodies. The images show respective autoradiograms of GroEL-UP (*upper panel*) and GroEL-P (*lower panel*). Multiple species of GroEL-P were observed indicating different levels of phosphorylation. **d** Gel filtration of purified GroEL-P and GroEL-UP. The graphs show presence of higher ratio of tetradecameric species in GroEL-P (*upper panel*) as compared with GroEL-UP (*lower panel*), which showed majority of dimer and heptamer. **e** Interaction of GroES with GroEL-P and GroEL-UP were analyzed by proteinase K-mediated partial cleavage. As compared with GroEL-UP:GroES, the phosphorylated GroEL-P:GroES complex was much more protected from protease cleavage indicating a stronger interaction. The experiment was performed thrice and the error bars show the SE of three independent readings

Since GroEL, a protein-folding chaperone, is essential for major cellular processes and development including biofilm formation in some bacteria,^31–33^ we wanted to further understand the molecular implications of PrkC-mediated GroEL phosphorylation. GroEL was expressed in the presence of either PrkC or PrpC in the surrogate host *Escherichia coli* (*E. coli*). The phosphorylation was confirmed by metabolic labeling with [^32^P]orthophosphoric acid (Fig. 4a) and Pro-Q Diamond staining (Fig. 4b). PrkC readily phosphorylated GroEL (GroEL-P), whereas no phosphorylation was observed in the presence of the corresponding phosphatase PrpC (GroEL-UP) (Fig. 4a, b). The stoichiometry of GroEL-P and GroEL-UP was analyzed by two-dimensional PAGE-based separation followed by immunoblotting with anti-GroEL antibodies. We found multiple isoforms generated after phosphorylation in GroEL-P and separated on the basis of their isoelectric points (Fig. 4c). In contrast, a single species of GroEL-UP was observed, indicating the lack of phosphorylation. These results are in agreement with above-mentioned status of GroEL phosphorylation during biofilm formation (Fig. 3b).

To understand the impact of GroEL phosphorylation, we analyzed the chaperonin function on the basis of structural organization of GroEL. The chaperone complex is formed by specialized tetradecameric GroEL rings that work in conjunction with a heptameric GroES cap.^34^ GroEL monomeric forms have negligible folding activity in vitro and the oligomeric structure of GroEL/GroES is required for biologically significant chaperonin function.^32^ We performed size exclusion chromatography of GroEL-P and GroEL-UP (using Superose 6 column) to determine GroEL complexes in both forms. Multimeric forms of GroEL-UP eluted as three peaks at 8.5, 13.5, and 15 ml, corresponding to tetradecamer (14-mer), heptamer (7-mer), and dimer (2-mer), respectively (Fig. 4d). Interestingly, maximum proportion of GroEL-P was found to be in the tetradecamer form. This indicated that phosphorylation of GroEL increases intermolecular interactions resulting in the formation of active tetradecamers. This observation also led us to speculate that PrkC-mediated phosphorylation might occur at the oligomerization interface of GroEL (equatorial domain).

### Effect of phosphorylation on GroES:GroEL interaction

The activity of GroEL is dependent on the successful interaction with the co-chaperone, GroES. To evaluate the effect of GroEL phosphorylation on its interaction with GroES, we utilized proteinase K resistance assay. In the absence of GroES, proteinase K preferentially cleaves GroEL at the accessible C-terminal region, resulting in a truncated protein of ~52 kDa. The compact double-ring cylindrical GroEL structure becomes more stable in the presence of GroES and is therefore protected from proteinase K-mediated proteolysis.^35^ We tested GroES-mediated protection of GroEL-P and GroEL-UP, and measured the uncleaved GroEL fraction. We observed that GroES protected the phosphorylated form of GroEL (53%) more than the unphosphorylated form (36%) from the proteinase K cleavage (Fig. 4e). This indicate that GroES:GroEL-P interaction is stronger than GroES:GroEL-UP, and therefore GroEL-P represents the active form of protein.

### Identification of phosphorylation sites in GroEL and their structural arrangement

**Fig. 5:**
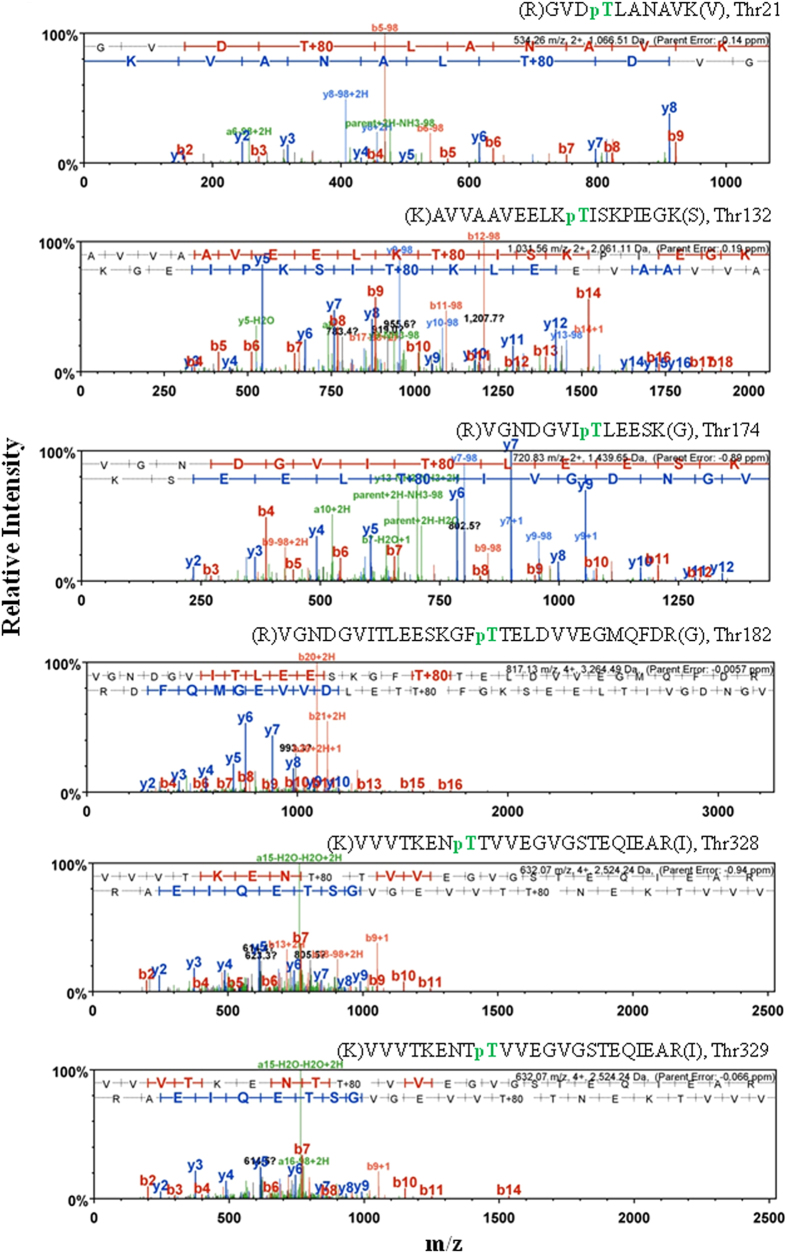
Phosphorylation sites of GroEL: mass spectrometry spectra showing the phosphorylation sites of GroEL. The spectra were displayed using the Scaffold software and corresponding trypsinized peptides are shown. Phosphorylated threonine residues within the identified peptides are marked in *green*

**Fig. 6:**
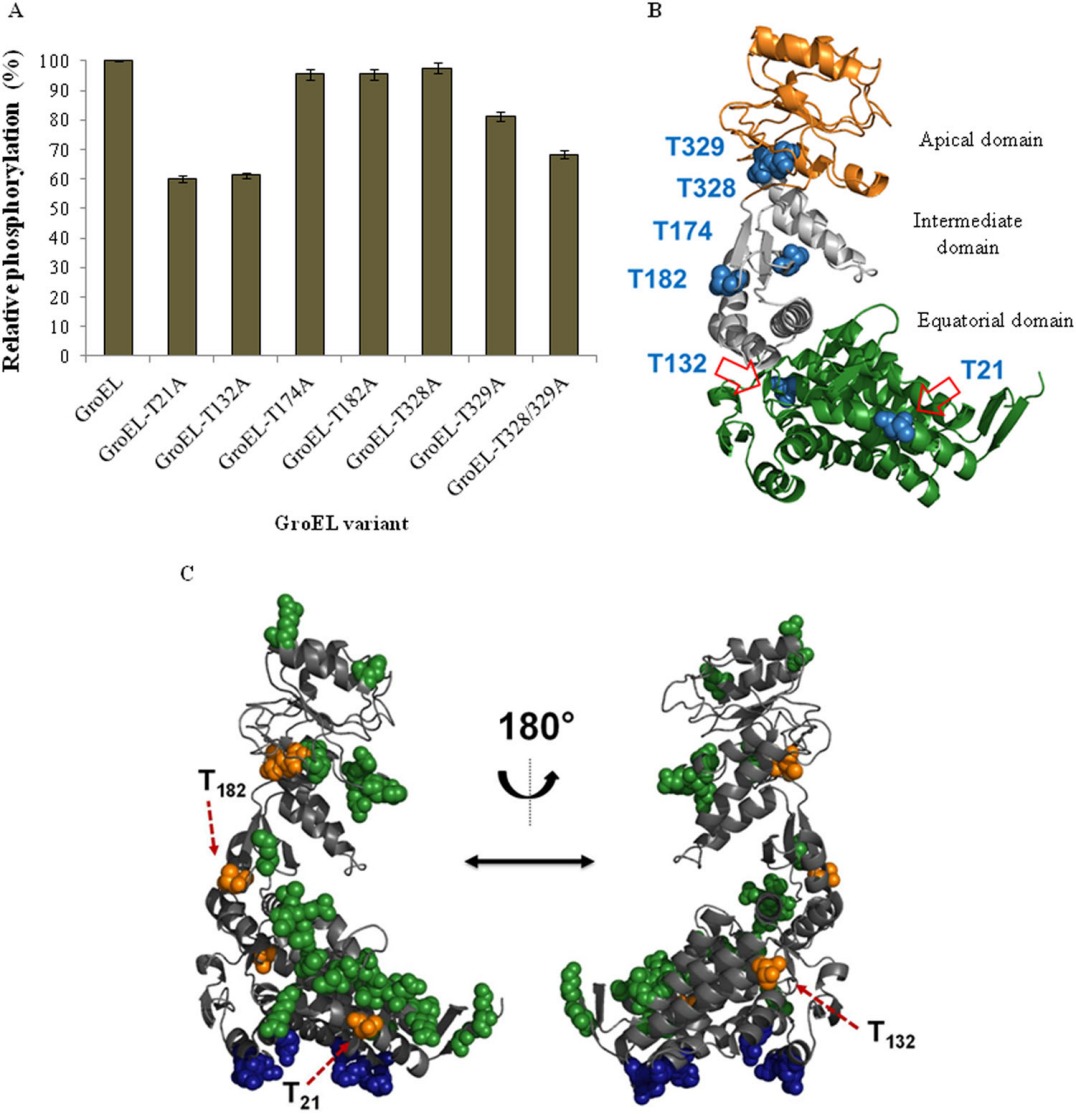
Structural localization of GroEL phosphorylation sites: **a** To understand the contribution of individual phosphorylation sites in GroEL, non-phosphorylatable mutants (Thr to Ala) were generated and were used for in vitro kinase assays with PrkC_c_. The band intensities on autoradiogram were estimated by QuantityOne (BioRad). Phosphorylation signal on native GroEL band was taken as 100% and relative phosphorylation was calculated in the remaining samples. The experiment was performed thrice and the error bars indicate the SE of three independent values. As clearly evident, major loss in phosphorylation was observed in GroEL-T21A, GroEL-T132A, and GroEL-T328/329A. **b** The structural model of GroEL was generated by Modeller v9.13 using the co-ordinates from *E. coli* GroEL structure (PDB code: 1AON chain A). The diagrammatic representation depicts three distinct regions in GroEL: apical (*gold*), intermediate (*silver*), and equatorial (*green*). The figures were generated in Pymol 1.3. Phosphorylated threonine residues are indicated as spheres in *blue*. The six phosphorylation sites have been marked in the respective regions. **c** Molecular model depicting the three-dimensional structure of *B. anthracis* GroEL monomer. The phosphothreonine residues (*broken red arrows*) possibly involved in multimerization have been marked. Molecular visualizations were performed using the Pymol Molecular Graphics System v1.3

Presence of multiple isoforms on two-dimensional PAGE led us to hypothesize that GroEL-P is phosphorylated on more than one amino acid residues (Fig. 3c). To validate this hypothesis, GroEL-P and GroEL-UP were subjected to mass spectrometry. The analysis identified six phosphorylated threonine residues in GroEL-P-Thr21, Thr132, Thr172, Thr184, Thr328, and Thr329 (Fig. 5). Mass spectrometry did not identify any phosphorylated residue in GroEL-UP, indicating the specificity of PrkC-mediated GroEL phosphorylation. To study the contribution of individual sites in GroEL phosphorylation, the non-phosphorylatable mutants were generated as—GroEL-T21A, GroEL-T132A, GroEL-T172A, GroEL-T184A, GroEL-T328A, GroEL-T329A, and the double mutant GroEL-T328/329A. The phosphorylation of GroEL was compared with each of these mutants. As shown in Fig. 6a, GroEL-T21A, GroEL-T132A, and GroEL-T328/329A showed maximum loss in phosphorylation compared with GroEL. These results suggest that Thr21, Thr132 and either of Thr328 or Thr329 are the major phosphorylation sites in GroEL.

Subsequently, we assessed the arrangement of phosphorylation sites in GroEL structure. Protein sequence of GroEL is highly conserved, with 64% identity to *E. coli* GroEL. On the basis of this similarity, we generated the three-dimensional structure of *B. anthracis* GroEL through homology modeling using Modeller v9.13 (Fig. 6b). The structure shows that two of the phosphorylation sites Thr328 and Thr329 lie in the apical domain of GroEL, Thr21, and Thr132 are present in the equatorial domain, and Thr174 and Thr182 are present in the intermediate domain (Fig. 6c). Since oligomerization of *B. anthracis* GroEL is influenced by phosphorylation (Fig. 4d), we tried to understand which residues play an important role in this regulation and thereby overall activity of *B. anthracis* GroEL.

### Complementation of the *groEL44* allele in *E. coli*

**Fig. 7:**
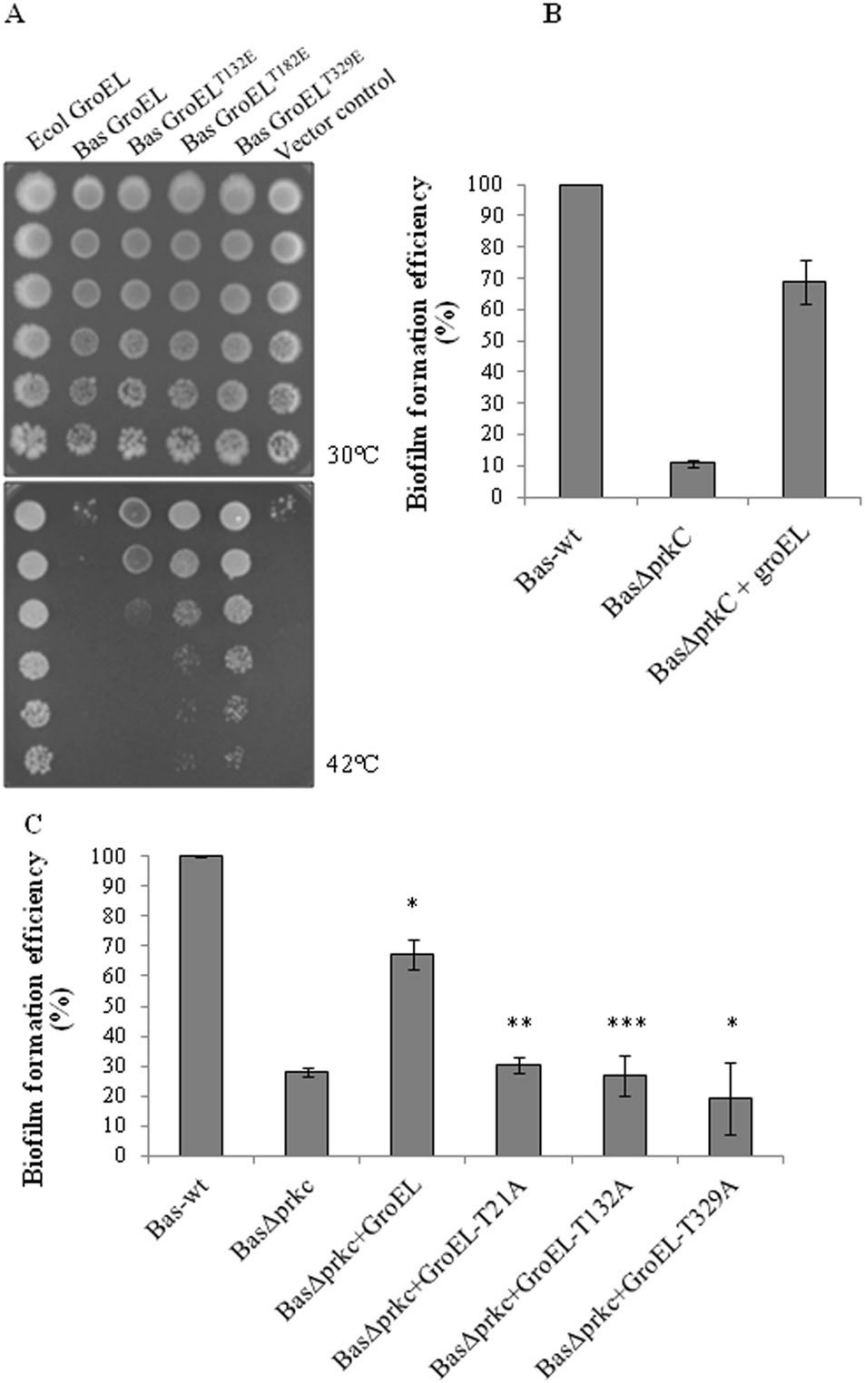
Role of GroEL phosphorylation sites: **a** *B. anthracis* GroEL and GroES were expressed in *E. coli* SV2 temperature sensitive strain and spotting was performed. To assay the effect of GroEL phosphorylation, specific phospho-mimetic mutants were also expressed at 30 °C (*upper panel*) and 42 °C (*lower panel*). All the strains grew similarly at permissive temperature (30 °C) but not at 42 °C. As compared with native GroEL, cells expressing phospho-mimetic mutants were able to grow at 42 °C. The images show serially diluted cells from *top* to *bottom* in both the panels. **b** The biofilm formation efficiencies of *Bas*-wt and *Bas*Δ*prkC* strains were compared with *Bas*Δ*prkC* cells overexpressing GroEL and calculated by crystal violet assay. As observed before (Fig. 1b), deletion of *prkC* led to loss of biofilm formation, which was recovered after overexpression of GroEL. Biofilm formation in *Bas*-wt was taken as 100% and relative efficiency was calculated. The experiment was performed thrice and the error bars indicate the SE of three independent values. **c** The biofilm formation efficiencies of *Bas*-wt and *Bas*Δ*prkC* strains were compared with *Bas*Δ*prkC* cells overexpressing either native GroEL or its phosphorylation site mutant (GroEL-T21A, GroEL-T132A, and GroEL-T329A), using crystal violet assay. The experiment was performed four times and the error bars show SE of four independent biological replicates. The *p*-value was calculated using two-tailed *t*-test, as shown by asterisk (*p < 0.05, **p ≤ 0.005, ***p ≤ 0.001)

To establish that phosphorylation is essential for GroEL activity in vivo, effect of GroEL phosphorylation was subsequently tested for complementation of *E. coli* GroEL. *E. coli* SV2 strain harbors a temperature-sensitive *groEL44* allele and cannot be grown at 42 °C unless expressing a functional GroEL.^36^ To understand the functionality of GroEL, we expressed *B. anthracis* GroEL along with its cognate GroES in *E. coli* SV2 at permissive (30 °C) and restrictive (42 °C) temperatures. The expression of *B. anthracis* GroEL and GroES did not lead to *E. coli* SV2 growth at 42 °C (Fig. 7a). Similarly, there was no growth when non-phosphorylatable mutants of GroEL (pThr to Ala) were expressed (data not shown). Considering the fact that *B. anthracis* GroEL is active in the phosphorylated form, we expressed the phospho-mimetic mutants (pThr to Glu) of selected sites that showed major loss of phosphorylation in GroEL (GroEL-Thr132, Thr182, and Thr329, Fig. 7a) in *E. coli* SV2. Interestingly, the cells expressing phospho-mimetic mutants were able to survive at restrictive temperature, whereas the wild-type GroEL could not survive (Fig. 7a). Of all the mutants, *B. anthracis* GroEL^T329E^ was most functional at the 42 °C and was able to complement *groEL44* more successfully.

### Phenotypic complementation of PrkC by GroEL

Our results establish that GroEL is an important substrate of PrkC and its structural stabilization and activity is dependent on PrkC-mediated phosphorylation. Since *prkC* deletion caused loss in biofilm formation (Fig. 1), and if it is mediated by the downstream substrate GroEL, the loss should get complemented by increasing GroEL concentration. To test this hypothesis, GroEL was overexpressed in *Bas*Δ*prkC* strain and its effect was analyzed on biofilm formation. Expressing GroEL at higher concentration led to a partial resumption of biofilm formation in *Bas*Δ*prkC* strain (Fig. 7b). This result showed that GroEL is required for biofilm formation in *B. anthracis* and its overexpression can compensate for the defect in PrkC.

To further understand the role of phosphorylated residues in GroEL on biofilm formation, we overexpressed each non-phosphorylatable GroEL mutant, GroEL-T21A, GroEL-T132A, and GroEL-T329A, in *Bas*Δ*prkC* and compared the biofilm formation with *Bas*-wt and *Bas*Δ*prkC*+*groEL* strains (Fig. 7c). As evident from the Fig. 7c, *Bas*Δ*prkC* strain did not form biofilm when GroEL phospho-ablative mutants were overexpressed, and thus were not able to compensate for the loss of PrkC as compared with native GroEL. This data indicated that Thr21, Thr132, and Thr329 are critical for the activity of GroEL, and any perturbation of these residues may influence GroEL activity, and consequently the biofilm-forming ability of *B. anthracis*.

### Phosphorylation of GroEL in other bacteria

**Fig. 8:**
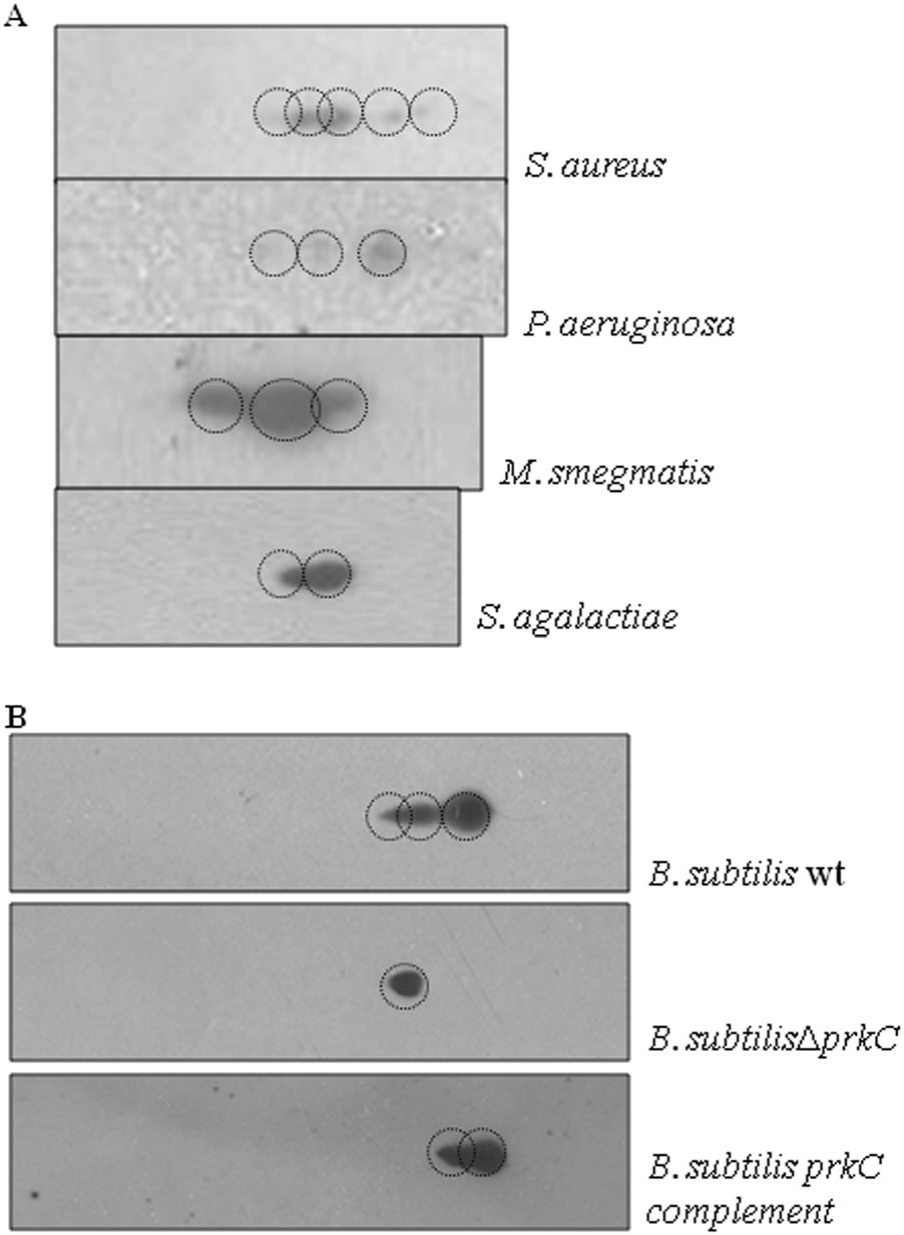
Conservation of GroEL phosphorylation in multiple bacteria: **a** The phosphorylation status of GroEL was studied in different bacterial species: *S. aureus*, *P. aeruginosa*, *M. smegmatis*, and *S. agalactiae*. Owing to its highly conserved sequence (Supplementary File), the cell lysates were probed with *B. anthracis* GroEL antibody, except *M. smegmatis*, which was probed with mycobacterial GroEL antibody. In all the strains studied, GroEL was separated into multiple isoforms, indicating the conservation of phosphorylation. **b** The specific phosphorylation of GroEL by PrkC was also studied in *B. subtilis* by using the *prkC* deletion strain and its complement. Three species of GroEL were observed in *B. subtilis* wild-type strain (*first panel*), which were lost in *B. subtilis*Δ*prkC* (*second panel*) and recovered by complementation of *prkC* (*third panel*)

PrkC homologs phosphorylate multiple substrates and have a role in virulence and bacterial development.^14, 15, 19, 21, 22, 27, 37–40^ To test the dependency of GroEL phosphorylation on PrkC in *B. subtilis*, a close relative of *B. anthracis*, we compared the phosphorylation status of GroEL by protein isoform analysis. On comparing the *B. subtilis* wild-type, *B. subtilis*Δ*prkC*, and *B. subtilis prkC* complemented strains, we observed that only a single species of GroEL was present in the *B. subtilis*Δ*prkC* strain whereas multiple isoforms were observed in the presence of PrkC (Fig. 8b). Thus, as in *B. anthracis*, GroEL is specifically phosphorylated by PrkC in *B. subtilis*. Since PrkC is a conserved Ser/Thr kinase that can get activated by sensing shared environmental cues, so bacteria may share common regulatory strategies involving PrkC and GroEL. To confirm this, we selected five diverse bacterial species that encode a chaperone with close homology to *B. anthracis* GroEL (Supplementary File, multiple sequence alignment). We prepared whole cell lysates of bacteria—*B. subtilis*, *Staphylococcus aureus* (*S. aureus*), *Streptococcus agalactiae* (*S. agalactiae*), *Pseudomonas aeruginosa* (*P. aeruginosa*), and *Mycobacterium smegmatis* (*M. smegmatis*)—and performed two-dimensional SDS-PAGE followed by immunoblotting with *B. anthracis* GroEL antibodies and *M. tuberculosis* GroEL2 antibodies. In this experiment, we detected multiple isoforms of GroEL in all these bacteria, possibly indicating the phosphorylated species (Fig. 8a). Thus, GroEL phosphorylation seems to be a conserved phenomenon; however, the sites of phosphorylation and the mechanistic details of such regulation still needs to be defined.

## Discussion

Many species of pathogenic bacteria are difficult to eradicate due to formation of resistant biofilms and spores. PrkC, a sensory STPK, and its homologs control the bacterial cell fate being the key regulator of cellular development and physiology. In *B. subtilis*, PrkC regulates spore formation as well as germination and biofilm formation,^41^ although the role of other signaling modules has also been shown under certain conditions.^42, 43^ Similar roles of PrkC in biofilm formation have been indicated in *Staphylococcal sp.* pathogens,^39, 44^ but the molecular mechanism remains unknown. PrkC also affects the pathogenicity of *Streptococcus mutans* through regulation of multiple processes, such as genetic transformability, cell shape and division, growth and stress response.^37, 45^ In this study, we have shown that PrkC plays a critical role in *B. anthracis* biofilm formation, with GroEL as the primary mediator. GroEL is an essential chaperone present in diverse bacteria that forms nanocages and provide central compartment to prevent aggregation of unfolded proteins.^46^ Employing biochemical and proteomic strategies, we found that GroEL is one of the most consistent substrate of *B. anthracis* PrkC, and this phosphorylation is conserved among other bacterial species.

*B. anthracis* GroEL is highly immunogenic and generates stronger immune response.^47^ Mice pre-injected with GroEL are protected against anthrax infection, indicating that GroEL might be important for pathogenesis.^48^ GroEL is co-transcribed with the co-chaperone GroES and the GroEL–GroES complex mediates appropriate protein folding. GroEL forms homo-heptamers and two such heptamers join to form a tetradecameric ring-like structure, which is assisted by a GroES heptameric cap.^32^ This whole complex mediates folding of unfolded or incorrectly folded peptides, resulting in a correctly folded and active protein. GroEL oligomerization is important for its protein-folding activity and possibly pathogenesis.^36^ Specifically, previous studies in *M. tuberculosis* and *E. coli* have shown that GroEL phosphorylation induces its multimerization, making it an active chaperone complex.^36, 49, 50^ We also found that GroEL-P is present in tetradecamer form as compared with unphosphorylated species. In addition, our results show that phosphorylated GroEL efficiently interacts with GroES, thus having a higher tendency to constitute an active complex. This indicates that phosphorylation facilitates the formation of active GroEL–GroES complex in *B. anthracis*.

The expression of GroEL is induced during stress conditions, such as high temperature, sporulation, or biofilm formation, indicating that GroEL is required for natural stress response of the cell.^31, 51, 52^ Furthermore, *B. anthracis* GroEL was found to be phosphorylated in biofilm-forming cells, indicating the necessity of the active protein for biofilm formation. Under these conditions, GroEL might promote the repair of damaged proteins or facilitate the folding of other overexpressed proteins. In addition, GroEL itself forms amyloid-like fibrils^53^ and it would be interesting if it has any direct physical role in biofilm organization. Although, GroEL is known to be involved in biofilm formation of a number of bacteria such as *Haemophilus influenzae*, *Campylobacter jejuni*, *S. mutans*, and mycobacteria,^30, 31, 54–56^ we, however, cannot rule out the possible existence of other PrkC substrate proteins that can influence the biofilm formation in co-operation with GroEL. PrkC and its homologs are shown to regulate multiple pathways by phosphorylation of specific proteins and it is possible that defect in biofilm formation is due to GroEL. Deletion of *prkC* results in unphosphorylated GroEL, which is less active, ultimately causing loss of biofilm formation in *B. anthracis*. Complementing the *prkC* deletion strain with GroEL partially regained its ability to form biofilms, indicating that an excess of unphosphorylated GroEL may partially supplement for phosphorylated active GroEL. To further confirm this phenomenon, we also complemented the *prkC* deletion strain with GroEL phosphorylation site mutants that were found to be inactive in *E. coli* SV2 complementation experiments. These mutants were not able to retrieve the biofilm formation in *Bas*Δ*prkC* strain, thus reaffirming our results.

Initiation of bacterial biofilm requires molecular factors for attachment and accumulation. In this complex, multifactorial process, both protein and DNA form biofilm matrix. The composition of the protein includes surface proteins and adhesins that form amyloid fibers. GroEL is often characterized as cell surface protein in gram-positive bacteria that overexpresses during biofilm formation.^57^ In fact *B. anthracis* GroEL has been reported to be present on the exosporium, cell surface, and secretome.^48^ GroEL is also known to bind to plasminogen and help in evading innate immune response.^58^ Opsonization of cell surface GroEL in *B. anthracis* leads to immunomodulation and protection in mice.^48^ This is the first study that connects the role of PrkC-mediated biofilm formation with GroEL phosphorylation. Being an abundant surface and secreted protein, GroEL nanocages can be a part of biofilm matrix or may aid in preventing proteostatis and aggregation.^46^ Future studies will be needed to define role of GroEL in biofilm formation and cell surface attachment in *B. anthracis*.

## Methods

### Bacterial strains and growth conditions

*E. coli* strain DH5α (Novagen) was used for cloning and BL21 (DE3) (Stratagene) was used for the expression of recombinant proteins. *E. coli* cells were grown as described before.^13, 21^ *B. anthracis* Sterne strain (wild-type *Bas*-wt and *Bas*Δ*prkC* ^17^), and *B. subtilis* were grown in LB broth at 37 °C with shaking at 200 rpm. For solid media, LB-Agar was used for both *E. coli* and *B. anthracis. S. agalactiae* was grown in BHI broth and agar at 37 °C. *S. aureus* (ATCC 29213) and *P. aeruginosa* (ATCC 25668) strains^59^ were maintained on Mueller Hinton broth and agar (Difco, Franklin Lakes, NJ, USA). *M. smegmatis* was grown in standard culture medium as described before.^13, 21, 17, 60^

### Biofilm formation in *B. anthracis* and crystal violet assay

*B. anthracis* Sterne strains (*Bas*-wt, *Bas*Δ*prkC*, *Bas*-*prkC*-comp, *Bas*Δ*prkC*+*groEL*, *Bas*Δ*prkC*+*groEL-T21A*, *Bas*Δ*prkC*+*groEL-T132A*, and *Bas*Δ*prkC*+*groEL-T329A*) were grown until late log phase and secondary cultures (0.01%) were inoculated in 6-well plates containing 5 ml LB media. The plates were incubated without shaking at 37 °C for 72 h and biofilms were observed. For quantitation of biofilms, crystal violet assay was performed as described before.^61^ Microscopic images were taken by an inverted microscope (Nikon Eclipse Ti, Nikon, Tokyo, Japan).

### *B. anthracis* lysate preparation

Cells were harvested from 50 ml logarithmic phase bacterial culture and washed twice with 1× PBS. Cells were resuspended in 5 ml of lysis buffer (PBS 1×, protease inhibitor cocktail [Roche], 1 mM PMSF, phosphatase inhibitor cocktail [Pierce], 1 mg/ml lysozyme, and 1 mM NaF) and sonicated for 10 min. The lysates were clarified and protein concentration was estimated by Bradford assay.

### Immunoprecipitation of phosphorylated proteins in *B. anthracis*

*B. anthracis* Sterne strain grown to an OD_600_ of ~1.0 in LB broth was harvested and suspended in lysis buffer containing 50 mM Tris-HCl [pH 7.5], 10% glycerol, 0.1% Triton X-100, 1× Protease inhibitor, and 1 mM PMSF and 50 ng/µl lysozyme. The suspended cells were incubated at 37 °C for 30 min and sonicated for 5 min. After sonication, the lysate was centrifuged at 15,000x*g* for 30 min and the supernatant (containing 10 mg protein) was incubated overnight at 4 °C with Protein A-Sepharose (Invitrogen, India) linked to either α-pSer or α-pThr antibodies. Immunoprecipitates were washed several times with 1% Triton X-100 in 10 mM Tris-Cl [pH 7.5] and the protein–antibody complex was eluted using Glycine elution buffer [pH 2.0]. The eluted immuno complexes were resuspended in 1× SDS sample buffer, resolved on SDS-PAGE, and analyzed by mass spectrometric analysis (UDSC, New Delhi) after staining with Coomassie Brilliant Blue R-250.

### Phosphoenrichment

400 μg of cell lysates (*Bas*-wt and *Bas*Δ*prkC*) were phospho-enriched by Phospho-Protein purification kit (Pierce), according to the manufacturer’s instructions. The enriched proteins were concentrated and equal amounts were resolved on SDS-PAGE. Differentially enriched proteins were identified by mass spectrometry.

### Identification of phosphorylation sites

To detect the phosphorylated proteins and peptides, the manually picked gel pieces were trypsinized and prepared for mass spectrometric analysis (Supplementary File).^62^

### Cloning and mutagenesis of *B. anthracis* genes and complementation in *B. anthracis* Sterne strain

Gene cloning and site directed mutagenesis was done using standard molecular biology procedures as described before^14, 22^ and *B. anthracis* Sterne strain genomic DNA. The clones were confirmed with restriction digestion and DNA sequencing (Invitrogen). Site-directed mutagenesis was carried out using QuikChange II XL Site-Directed Mutagenesis Kit (Stratagene) using GroEL clones as templates. The details of primers and plasmids are provided in Table S1.

Genes encoding for PrkC (1-657 aa), wild-type GroEL (1-544 aa), and GroEL mutants (GroEL-T21A, GroEL-T132A, and GroEL-T329A) were cloned into the *E. coli*, *B. anthracis* shuttle vector, pYS5 (modified to express Spectinomycin resistance gene^63^), and electroporated in *B. anthracis* Sterne strain (*Bas*-wt or *Bas*Δ*prkC* strain) (BTX Electro Cell Manipulator 600).

### GroEL complementation in *E. coli* SV2 strain

Complementation study on *B. anthracis* GroEL variants was performed as described previously.^36^ Briefly, *B. anthracis groEL* variants and *groES* were cloned into pProEx-HTc and cloned in pACYCDuet-1, respectively. Individual *groEL* variants were co-expressed with *groES* in *E. coli* SV2. The cultures of *E. coli* SV2 expressing the required genes were serially diluted and spotted onto LB agar plates. The plates were incubated at permissive (30 °C) and restrictive (42 °C) conditions. Plasmid encoding *E. coli* GroES and GroEL, pSCM1603, was included as control.^36^

### Expression and purification of recombinant proteins from *E. coli*

The recombinant plasmids were transformed and proteins were overexpressed in *E. coli* BL-21 (DE3). The recombinant GST-tagged and His_6_-tagged fusion proteins were affinity purified with glutathione sepharose column (Qiagen, India) and Ni^2+^-NTA affinity column (Qiagen), respectively, as described previously.^64^

### In vitro kinase and phosphatase assays

In vitro kinase assays (PrkC kinase/catalytic domain PrkC_c_, 1 μg) were carried out in kinase buffer (20 mM HEPES pH 7.2, 10 mM MgCl_2_, and 10 mM MnCl_2_) containing 2 μCi [*γ*-^32^P]ATP (BRIT, Hyderabad, India) followed by incubation at 25 °C for 30 min or as indicated in the text. Phosphorylation assays of substrates were carried out similarly, using 5 μg substrates (Ef-Tu, Ef-G, SodA2, and GroEL). Reactions were terminated by 5× SDS sample buffer followed by boiling at 100 °C for 5 min. Proteins were separated by SDS-PAGE and analyzed by Personal Molecular Imager (PMI, BioRad). The images were quantitated by QuantityOne^®^ software (PMI, BioRad). Dephosphorylation was carried out by incubating the kinase reaction samples with Ser/Thr phosphatase PrpC (1 μg) for additional 30 min at 37 °C, as described.^14, 22^

For time-dependent kinase assay, PrkC_c_ was first autophosphorylated (preactivated) using cold ATP. The autophosphorylated active kinase was then incubated with GroEL or SodA2 in kinase buffer containing 2 μCi [*γ*-^32^P]ATP with increasing time points up to 30 min. The phosphotransfer on substrates was observed and quantitated.

### Immunoblotting to identify phosphorylated residues

To detect the phosphorylated proteins, immunoblotting with *α*-pThr was performed as described previously.^13^ Proteins were resolved by SDS-PAGE and transferred onto a nitrocellulose membrane. Blots were exposed to *α*-pThr antibody and goat anti-rabbit IgG secondary antibodies. The blots were developed by SuperSignal® West Pico Chemiluminescent Substrate kit (Pierce Protein Research Products), according to the manufacturer’s instructions.

### Co-expression of GroEL with PrkC and PrpC

*Bas0253* cloned in pGEX-5X-3 or pProEx-HTc was co-expressed in *E. coli* BL-21 (DE3) cells with pACYC-PrkC or pACYC-PrpC, to generate phosphorylated and unphosphorylated proteins, respectively. The co-expressed transformants were selected on ampicillin and chloramphenicol. The selected colonies were grown and maintained in media containing ampicillin and chloramphenicol and proteins were overexpressed with Isopropyl β-D-1-thiogalactopyranoside (IPTG). Phosphorylation status of these proteins was analyzed by Pro-Q^®^ Diamond phospho-specific stain (Molecular Probes, Life Technologies) followed by SYPRO^®^ Ruby Protein Gel stain (Molecular Probes, Life Technologies) and Coomassie Brilliant Blue stain, according to the manufacturer’s instructions. Pro-Q Diamond is a sensitive non-covalent fluorescent dye staining technology used for the detection of phosphoserine, phosphothreonine, and phosphotyrosine containing proteins. These proteins were used for subsequent assays.

### Recombinant GroEL size exclusion chromatography

His_6_-tagged GroEL-UP or GroEL-P were used for purification by size exclusion chromatography. Similar procedure was followed as described earlier.^36^ Briefly, the Ni^2+^-NTA purified proteins were dialyzed with 50 mM Tris (pH 8.0), 150 mM NaCl, and 1 mM EDTA. The preparations were resolved on Superose 6 10/100 GL (GE Healthcare) connected to the NGC Quest Plus Chromatography System (BioRad), with column volume of 23.6 ml and flow rate of 0.5 ml/min. The protein standards on Superose 6 column were: Thyroglobulin (660 kD), Ferritin (440 kD), BSA (66 kD), and RNase A (13.7 kD) that elute at 12, 14, 16.5, and 18 ml, with partition coefficients (*K*_av_) of 0.28, 0.40, 0.56, and 0.72, respectively.

### Proteinase K protection assay

Protection of GroEL by GroES was performed as described earlier with minor modifications.^65^ His_6_-tagged GroEL-UP and GroEL-P (0.8 µM each) were incubated for 10 min at 25 °C in buffer A (10 mM MOPS-KOH [pH 7.2] and 50 mM KCl) containing 5 mM Mg-acetate, 1 mM ADP, and 12.5 µM His_6_-tagged GroES. The reactions were then cooled to 0 °C and incubated with proteinase K (12.5 µg/ml). At times 0 and 2 min, aliquots were removed and PMSF was added to a final concentration of 1 mM. The samples were then subjected to TCA precipitation and precipitates were resolved by SDS-PAGE and analyzed by immunoblotting using anti-GroEL antibodies.

### GroEL structure generation

*B. anthracis* GroEL sequence was modeled using the co-ordinates from *E. coli* GroEL structure (PDB code: 1AON chain A) with Modeller v9.13. The figures were generated in Pymol 1.3 as described earlier.^66^ Phosphorylated threonine residues are indicated as spheres in *blue*. Apical, intermediate, and equatorial domains are color coded in *gold*, *silver*, and *green*, respectively.

Few methods have been discussed in detail in Supplementary File.

## Electronic supplementary material

Supplementary Information

Supplementary Figure 1

Supplementary Figure 2

Supplementary Figure 3
