# Supplementary material for: Ser/Thr protein kinase PrkC-mediated regulation of GroEL is critical for biofilm formation in Bacillus anthracis
Source: NPJ Biofilms Microbiomes. 2017 Mar 7;3:7. doi: 10.1038/s41522-017-0015-4 (PMC5460178; doi:10.1038/s41522-017-0015-4)
Supplement: Supplementary file 1 — Supplementary Information [file 41522_2017_15_MOESM1_ESM.pdf]

Ser/Thr protein kinase PrkC mediated regulation of GroEL is critical for biofilm formation in *Bacillus anthracis*

Running title: *PrkC-GroEL interaction regulates biofilm formation*

Table S1: Primers and clones

**Genes**	**Vectors**	**Restriction sites**	**Primers (5′→3′)**[a]	**Reference**
prkC$prkC_c$ (*bas3713*, 1-1011 bp), kinase domain	pProEx-HTc	FP-BamHI	TAGGTGAAGTGGATCCTGCTGATTGGAAAACGC	[1]
		RP-XhoI	TGTAATTAAAATCTCGAGTCATTTATTACTTCGTTTG	
tuf (*bas0108*)	pGEX-5X-3	FP-BamHI	CCTATATAAACTAAGGAGGGGATCCGAATGGCTAAAGC	[1]
		RP-XhoI	GGGTTTTTATATCACTCGAGATTACTCAACGATAGTAGC	
prpC (*bas3714*)	pProEx-HTc	FP-BamHI	GCGCAAAGAAGAGACGAGGGATCCAGATGAAAGCCGTGTTTCT	[1]
		RP-XhoI	CGTTTTCCAATCAGCACGCTCGAGTTCACCTACTTTCGTTTGTCG	
$prkC_c^{K40M}$	pProEx-HTc	FP	CGGGATGTAGCGGTGATGATATTAAGACTCGAC	[1]
		RP	GTCGAGTCTTAATATCATCACCGCTACATCCCG	
fusA (*bas0107*)	pGEX-5X-3	FP-BamHI	CCATATATGGAAGGAGCAAGGATCCAAATGGCAAGAG	This study
		RP-XhoI	CGTGAAGAATCGATAAAAATCAATTCTCGAGCTTTATTTTTTTG	
sodA2 (*bas5300*)	pGEX-5X-3	FP-BamHI	GGGTGAAAGGAGGGATCCTCATGTCTTCATTTCA	This study
		RP- XhoI	CTTACCTTATACGCTCGAGCTAATGTTTTTGTG	
groEL (*bas0253*)	pGEX-5X-3, pProEx-HTc	FP-BamHI	AATCCAAGGGGGTGGATCCTTATGGCAAAAG	This study
		RP-XhoI	TTAGGGCAAACTCGAGTTACATCATTCCGCCC	
groES (*bas0252*)	pGEX-5X-3, pProEx-HTc	FP-BamHI	AAC AAA ATG AGG AGG ATC CTG TTC ATG CTA AAG	This study
		RP-XhoI	CCTTGGATTTCTCGAGATTTATATAATTAACCG	
	pACYCDuet-1	FP-NdeI	GAGGAGGTTATTGCATATGCTAAAGCCATTAGGTGATCG	This study
		RP-XhoI	CCTTGGATTTCTCGAGATTTATATAATTAACCG	
prkC-ys	pYS5	FP-SpeI	CCACTAGTCGTGCTGATTGGAAAACGCTTAAATG	This study
		RP-BamHI	CCGGATCCTTATTGTGTTGGATATGGTACTTCTTTG	

Duet-prkC	pACYCDuet-1	FP-NdeI	GGGTTCGACAAACGAAAGCATATGAAGTGCAACGTGCTG	This study
		RP-XhoI	CTCTAGAAAGAAACTCGAGTGTATTCTTCTTGTGTTGG	
groEL-ys	pYS5	FP-SpeI	CCAAGGGGGTCAACTACTAGTTATGGCAAAAGATATTAA	This study
		RP-BamHI	GGGCAAACGGATCCATTACATCATTCCGCCCATACC	
groEL^{T21A}	pProEx-HTc, pYS5	FP	CGATGCTTCGCGGTGTCGACGCTCTTGCAAATGCAGTAAAAG	This study
		RP	CTTTTACTGCATTTGCAAGAGCGTCGACACCGCGAAGCATCG	
groEL^{T132A}	pProEx-HTc, pYS5	FP	GCAGTAGAAGAATTAAAAGCGATTTCTAAACCAATCG	This study
		RP	CGATTGGTTTAGAAATCGCTTTTAATTCTTCTACTGC	
groEL^{T174A}	pProEx-HTc	FP	GGTAACGACGGCGTTATTGCTTTAGAAGAATCTAAAGG	This study
		RP	CCTTTAGATTCTTCTAAAGCAATAACGCCGTCGTTACC	
groEL^{T182A}	pProEx-HTc	FP	GAAGAATCTAAAGGTTTCGCAACAGAATTAGACGTAG	This study
		RP	CTACGTCTAATTCTGTTGCGAAACCTTTAGATTCTTC	
groEL^{T328A}	pProEx-HTc	FP	GTTGTAACGAAAGAAAACGCAACTGTAGTTGAAGGTG	This study
		RP	CACCTTCAACTACAGTTGCGTTTTCTTTCGTTACAAC	
groEL^{T329A}	pProEx-HTc, pYS5	FP	GTAACGAAAGAAAACACAGCTGTAGTTGAAGGTGTAG	This study
		RP	CTACACCTTCAACTACAGCTGTGTTTTCTTTCGTTAC	
groEL$^{T328/329A}$	pProEx-HTc	FP	GTTGTAACGAAAGAAAACGCAGCTGTAGTTGAAGGTGTAGG	This study
		RP	CCTACACCTTCAACTACAGCTGCGTTTTCTTTCGTTACAAC	
groEL^{T132E}	pProEx-HTc	FP	GCAGTAGAAGAATTAAAAGAGATTTCTAAACCAATCG	This study
		RP	CGATTGGTTTAGAAATCTCTTTTAATTCTTCTACTGC	
groEL^{T182E}	pProEx-HTc	FP	GAAGAATCTAAAGGTTTCGAAACAGAATTAGACGTAG	This study
		RP	CTACGTCTAATTCTGTTTCGAAACCTTTAGATTCTTC	
groEL^{T329E}	pProEx-HTc	FP	GTAACGAAAGAAAACACAGAGGTAGTTGAAGGTGTAG	This study
		RP	CTACACCTTCAACTACCTCTGTGTTTTCTTTCGTTAC	
groEL-His	pYS5	FP-SpeI	CCAAGGGGGTCAACTAGTTATGGCAAAAGATATTAA	This study

		RP-BamHI	GGATCCGTGGTGATGGTGGTGGT GCATCATTCCGCCCATACC	

[a] restriction/mutation sites have been underlined

SUPPLEMENTARY METHODS

Biofilm formation in *B. anthracis* and crystal violet assay- *B. anthracis* Sterne strains (*Bas*-wt, *Bas*Δ*prkC*, *Bas-prkC*-comp, *Bas*Δ*prkC+groEL*, *Bas*Δ*prkC+groEL-T21A*, *Bas*Δ*prkC+groEL-T132A* and *Bas*Δ*prkC+groEL-T329A*) were grown until late log phase and secondary cultures (0.01%) were inoculated in 6-well plates containing 5 ml LB media. The plates were incubated without shaking at 37°C for 72 h and biofilms were observed. The culture supernatant was aspirated and adherent biofilms were washed twice with PBS. 1% crystal violet solution was added to the cells (1 ml each) and allowed to bind for 10 min at room temperature [2]. Excess solution was aspirated and washed twice with Phosphate Buffer Saline (PBS). The remaining stained biofilm was dissolved in 95% ethanol for 10 min and absorbance was taken at 600 nm. Culture media without any bacteria was used as a control. Microscopic images were taken by an inverted microscope (Nikon Eclipse Ti, Nikon, Tokyo, Japan).

Two-dimensional gel electrophoresis- Whole cell lysates of *Bas*-wt and *Bas*Δ*prkC* (25 µg each) were clarified by methanol chloroform extraction [3]. Precipitated samples were re-dissolved in rehydration buffer (7 M Urea, 2 M Thiourea, 2% CHAPS) containing 1% Biolyte ampholyte and 0.5% bromophenol blue. Rehydration of IPG strips (BioRad), pH range 4-7, was done overnight and focusing was performed. The strips were washed with equilibration buffer (6 M Urea, 0.375 M Tris-HCl pH 8.8, 2% SDS, 20% Glycerol) containing 2% DTT and 2.5% iodoacetamide. Second dimension was run in 10% SDS-PAGE followed by western blotting on nitrocellulose membrane. The blots were developed by anti-GroEL antibody. This method has been previously used for characterization and separation of phosphorylated proteins [4-6]. The gels were stained using Pro-Q Diamond stain followed by SYPRO Ruby stain. The gels were analyzed by Typhoon FLA 7000 imager (GE healthcare life sciences).

Mass spectrometry analysis- Samples were run on SDS-PAGE and in gel digestion was performed to extract the peptides from gel. Gel slices were washed with 1 ml LC-MS water, followed by vortexing for

5 min. The vials were spun 4-5 times to drain the excess water. 500 mM ammonium bicarbonate (ABC) and acetonitrile (ACN) (Sigma) were added to destain the gel followed by vortexing for 5 min. Destaining was performed overnight at 4°C shaking. Supernatant was removed after destaining and samples were washed with LC-MS water. 500 µl ACN was added for dehydration and gel slices were dehydrated with speed vac. Trypsinization was peformed with 50 ng Trypsin Gold (mass spec grade part no.-V528A, Promega) followed by incubation at 37°C for overnight. Peptide extraction was done subsequently by adding 10 µl of solution-1 (500 µl MilliQ water, 500 µl acetonitrile, 1 µl trifluoro acetic acid) followed by vortexing for 10 min and aspirating the aqueous solution. Peptide desalting was done with C18 resin tips or ZIPtips (Millipore). The peptides thus extracted were analyzed by MALDI (4800 PLUS MALDI TOF/TOF from AB SCIEX, Protein pilot software for data analysis, CIF, UDSC, New Delhi).

Identification of phosphorylation sites- To detect the phosphorylated proteins and peptides, the manually picked gel pieces were trypsinized and prepared for mass spectrometric analysis [7]. Peptides were separated and measured by LC-ESI-mass spectrometry using the Easy-nLCII HPLC system (Thermo Fisher Scientific, Waltham, MA) coupled directly to an LTQ Orbitrap VelosTM mass spectrometer (Thermo Fisher Scientific). The Easy-nLCII was equipped with a self-packed analytical column (C18-material [Luna 3u C18(2)100A, Phenomenex®], 100 µm I.D. × 200 mm column). Peptide elution was performed by application of a binary gradient from 5% buffer A (0.1% (v/v) acetic acid) to 75% buffer B (99.9% (v/v) acetonitrile, 0.1% (v/v) acetic acid) over a period of 45 min with a flow rate of 300 nl/min.

The LTQ Orbitrap Velos and Orbitrap XL were operated in the data-dependent MS/MS mode. The full scan was recorded in the Orbitrap analyzer at resolution R = 60,000, with wideband activation and lockmass option (enabled for the 445.120025 ion) activated. The use of m/z values as masses was enabled. Collision-induced dissociation (CID) spectra in the LTQ analyzer were logged for the 20 (Velos) or five (Orbitrap XL) most intense precursor ions. Multistage activation (MSA) at -97.98 Thompson (for H3PO4- loss at serine or threonine) was applied in all MS/MS events.

Proteins were identified by searching all MS/MS spectra against a forward reverse database that was composed of all protein sequences of *B. anthracis* Sterne and common contaminants using SorcererTM-SEQUEST® (Thermo Fisher Scientific, San Jose, CA, USA; version v.27, rev.11) in conjunction with Scaffold (version Scaffold 3 00 06, Proteome Software Inc., Portland, OR). Full tryptic specificity was assumed and up to two mis-cleavages were allowed. The maximum mass deviation of the precursor ions was set to 10 ppm and for the fragment ions to 1 Da. Methionine oxidation (+15.99492 amu), cysteine carbamidomethylation (+57.021465 amu) and phosphorylation (+79.966331 amu) at serine, threonine or tyrosine were set as variable modifications. Proteins were identified by applying a stringent SEQUEST filter. SEQUEST identifications required at least deltaCn scores exceeding 0.10 and XCorr scores exceeding 2.2, 3.3 and 3.75 for doubly, triply and quadruply charged peptides, respectively. A minimum of two peptides were needed for identification.

Expression and purification of recombinant proteins from *E. coli*- The recombinant plasmids were transformed and proteins were over-expressed in *E. coli* BL-21 (DE3). The recombinant GST-tagged and His_6-tagged fusion proteins were affinity purified with glutathione sepharose column (Qiagen, India) and Ni^{2+}-NTA affinity column (Qiagen), respectively, as described previously [8]. Purified proteins were resolved by SDS-PAGE and analyzed after staining with Coomassie Brilliant Blue R-250. Concentrations of the purified proteins were estimated by Bradford assay (Bio-Rad, India).

***In vitro* kinase and phosphatase assays-** *In vitro* kinase assays of the full-length kinase and its catalytic domain (1 μg) were carried out in kinase buffer (20 mM HEPES pH 7.2, 10 mM $MgCl_2$ and 10 mM $MnCl_2$) containing 2 μCi [γ-^{32}P]ATP (BRIT, Hyderabad, India) followed by incubation at 25°C for 30 min or as indicated in the text. Phosphorylation assays of substrates were carried out similarly, using 5 μg substrates (Ef-Tu, Ef-G, SODA2 and GroEL). Reactions were terminated by 5X SDS sample buffer followed by boiling at 100°C for 5 min. Proteins were separated by SDS-PAGE and analyzed by Personal Molecular Imager (PMI, BioRad). The images were quantitated by QuantityOne® software (PMI, BioRad). Dephosphorylation was carried out by incubating the kinase reaction samples with Ser/Thr phosphatase PrpC (1 μg) for additional 30 minutes at 37°C, as described[1, 9].

Immunoblotting to identify phosphorylated residues- To detect the phosphorylated proteins, immunoblotting with α-pThr was performed as described previously [10]. Proteins were resolved by SDS-PAGE and transferred onto a nitrocellulose membrane. Blots were then blocked with 3% bovine serum albumin (Sigma) in PBS containing 0.1% Tween 20 (PBST) overnight at 4°C. This was followed by incubation with α-pThr antibody (Invitrogen) at 1:10,000 dilution and goat anti-rabbit IgG secondary antibodies (Bangalore Genei) at 1:10,000 dilution for 1 h each at room temperature. The blots were developed by SuperSignal® West Pico Chemiluminescent Substrate kit (Pierce Protein Research Products) according to manufacturer's instructions.

SUPPLEMENTARY FIGURE LEGENDS

Figure S1. PrkC specific substrates: *Bas*-wt and *Bas*Δ*prkc* cell lysates were separated by 2D-PAGE using 4-16% gradient gels and stained with ProQ phospho-specific staining (upper panels). More phosphorylated bands were observed in the *Bas*-wt lysate as compared with *Bas*Δ*prkc* lysate, indicating the possible PrkC-specific targets. These proteins were subsequently compared by staining the gels with SyproRuby stain (lower panels). In upper ProQ stained panels, the bands that were more intense (blue numbered, PrkC influenced) or new (black numbered, PrkC specific) in *Bas*-wt as compared with *Bas*Δ*prkc* were analyzed by mass-spectrometry (Table 3). The bands are also encircled with corresponding color.

Figure S2. Time-dependent phosphorylation: Time-dependent phosphorylation of GroEL (A) and SODA2 (B), using autophosphorylated kinase (using cold ATP). Phosphorylation intensity at 30 minute time point was taken as 100% and relative phosphorylation was calculated followed by normalization with the protein amount (Figure 2). Upper panel shows autoradiograms and lower panel are corresponding coomassie stained SDS PAGE.

Figure S3. *In vitro* phosphorylation of PrkC substrates: To validate the identified PrkC substrates (Tables 1, 2 and 3), we used SODA2 (24 kDa), Ef-Tu (42.9 kDa) and Ef-G (76.3 kDa) for *in vitro* kinase assays using the kinase domain of PrkC [$PrkC_c$, 1-337 aa, ~40 kDa, [1]]. Autoradiograms show *in vitro*

phosphorylation of (A) Ef-Tu, (B) Ef-G, and (C) SODA2. PrkC catalytic domain ($PrkC_c$, 1 µg) was used for phosphorylation (for 30 min) of purified substrates (5 µg each) and the kinase dead mutant ($PrkC_c$-K40M) was used as a negative control. No phosphorylation was observed in the control reactions when the kinase inactive mutant $PrkC_c$-K40M was used [1].

Multiple Sequence Alignment: The protein sequences of GroEL from different species of bacteria were aligned using CLUSTAL multiple sequence alignment by MUSCLE (3.8). The bacterial species are as follows: Pae, *Pseudomonas aeruginosa*; Msm, *Mycobacterium smegmatis*; Sag, *Streptococcus agalactiae*; Sau, *Staphylococcus aureus*; Bas, *Bacillus anthracis* Sterne; Bsu, *Bacillus subtilis*. The protein sequences were extracted from NCBI.

```
Pae_Gro         MAAKEVKFGDSARKKMLVGVNVLADAVKATLGPKGRNVVLDKSFGAPTITKDGVSVAKEI
Msm_Gro         -MAKTIAYDEEARRGLERGLNSLADAVKVTLGPKGRNVVLEKKWGAPTITNDGVSIAKEI
Sag_Gro         -MAKDIKFSADARSAMVRGVDILADTVKVTLGPKGRNVVLEKAFGSPLITNDGVTIAKEI
Sau_Gro         -MAKQLKFSEDARQAMLRGVDQLANAVKVTIGPKGRNVVLDKEFTAPLITNDGVTIAKEI
Bas_Gro         -MAKDIKFSEEARRSMLRGVDTLANAVKVTLGPKGRNVVLEKKFGSPLITNDGVTIAKEI
Bsu_Gro         -MAKEIKFSEEARRAMLRGVDALADAVKVTLGPKGRNVVLEKKFGSPLITNDGVTIAKEI
                  ** : :. .**  :  *:: **::**.*:*********:* : :* **:***::****

Pae_Gro         ELKDKFENMGAQLVKDVASKANDAAGDGTTTATVLAQAIVNEGLKAVAAGMNPMDLKRGI
Msm_Gro         ELEDPYEKIGAELVKEVAKKTDDVAGDGTTTATVLAQALVREGLRNVAAGANPLGLKRGI
Sag_Gro         ELEDHFENMGAKLVSEVASKTNDIAGDGTTTATVLTQAIVREGLKNVTAGANPIGIRRGI
Sau_Gro         ELEDPYENMGAKLVQEVANKTNEIAGDGTTTATVLAQAMIQEGLKNVTSGANPVGLRQGI
Bas_Gro         ELEDAFENMGAKLVAEVASKTNDVAGDGTTTATVLAQAMIREGLKNVTAGANPMGLRKGI
Bsu_Gro         ELEDAFENMGAKLVAEVASKTNDVAGDGTTTATVLAQAMIREGLKNVTAGANPVGVRKGM
                **:* :*::**:** :**.*::: **********:**::.***. *::* **:.:..*:

Pae_Gro         DKATVAIVAQLKELAKPCADTKAIAQVGTISANSDESIGQIIAEAMEKVGKEGVITVEEG
Msm_Gro         EKAVEKVTETLLKSAKEVETKEQIAATAGISA-GDQSIGDLIAEAMDKVGNEGVITVEES
Sag_Gro         ETAVSAAVEELKEIAQPVSGKEAIAQVAAVSS-RSEKVGEYISEAMERVGNDGVITIEES
Sau_Gro         DKAVKVAVEALHENSQKVENKNEIAQVGAISA-ADEEIGRYISEAMEKVGNDGVITIEES
Bas_Gro         EKAVVAAVEELKTISKPIEGKSSIAQVAAISA-ADEEVGQLIAEAMERVGNDGVITLEES
Bsu_Gro         EQAVAVAIENLKEISKPIEGKESIAQVAAISA-ADEEVGSLIAEAMERVGNDGVITIEES
                : *.      *   ::    .. ** .. :*:  .:.:*  *:***:.**::****:**.

Pae_Gro         SGLENELSVVEGMQFDRGYLSPYFVNKPDTMAAELDSPLLLLVDKKISNIREMLPVLEAV
Msm_Gro         NTFGLQLELTEGMRFDKGYISGYFVTDAERQEAVLEDPYILLVSSKVSTVKDLLPLLEKV
Sag_Gro         RGMETELEVVEGMQFDRGYLSQYMVTDNDKMVSELENPYILITDKKISNIQEILPLLEEV
Sau_Gro         NGLNTELEVVEGMQFDRGYQSPYMVTDSDKMVAELERPYILVTDKKISSFQDILPLLEQV
Bas_Gro         KGFTTELDVVEGMQFDRGYASPYMITDSDKMEAVLDNPYILITDKKISNIQEILPVLEQV
Bsu_Gro         KGFTTELEVVEGMQFDRGYASPYMVTDSDKMEAVLDNPYILITDKKITNIQEILPVLEQV
                  :  :*.:.***.**.** * *::.. :   : *: * :*:...*::...::**:** *

Pae_Gro         AKAGRPLLIVAEDVEGEALATLVVNNMRGIVKVAAVKAPGFGDRRKAMLQDIAILTGGTV
Msm_Gro         IQSGKPLLIIAEDVEGEALSTLVVNKIRGTFKSVAVKAPGFGDRRKAMLQDMAILTGGQV
Sag_Gro         LKTNRPLLIIADDVDGEALPTLVLNKIRGTFNVVAVKAPGFGDRRKAMLEDIAILTGGTV
Sau_Gro         VQSNRPILIVADEVEGDALTNIVLNRMRGTFTAVAVKAPGFGDRRKAMLEDLAILTGAQV
Bas_Gro         VQQGKPLLIIAEDVEGEALATLVVNKLRGTFNVVAVKAPGFGDRRKAMLEDIAILTGGEV
```

```
Bsu_Gro          VQQGKPLLLIAEDVEGEALATLVVNKLRGTFNAVAVKAPGFGDRRKAMLEDIAVLTGGEV
                  : ..*:*::*::*:*:**..:*:*.:** .. .***************:*:*:***. *

Pae_Gro          ISEEVGLSLEGATLEHLGNAKRVVINKENTTIIDGAGVQADIEARVLQIRKQIEETTSDY
Msm_Gro          ISEEVGLSLETADVSLLGKARKVVVTKDETTIVEGAGDAEAIQGRVAQIRAEIENSDSDY
Sag_Gro          VTEDLGLDLKDATMQVLGQSAKVTVDKDSTVIVEGAGDSSAIANRVAIIKSQMEATTSDF
Sau_Gro          ITDDLGLDLKDATIDMLGTASKVEVTKDNTTVVDGDGDENSIDARVSQLKSQIEETESDF
Bas_Gro          ITEELGRDLKSATVESLGRAGKVVVTKENTTVVEGVGSTEQIEARIGQIRAQLEETTSEF
Bsu_Gro          ITEDLGLDLKSTQIAQLGRASKVVVTKENTTIVEGAGETDKISARVTQIRAQVEETTSEF
                 :::::* .*: : :  ** : .* : *:.*.:::* *    *  *:  :. ::* : *::

Pae_Gro          DREKLQERLAKLAGGVAVIKVGAATEVEMKEKKARVEDALHATRAAVEEGVVPGGGVALV
Msm_Gro          DREKLQERLAKLAGGVAVIKAGAATEVELKERKHRIEDAVRNAKAAVEEGIVAGGGVALL
Sag_Gro          DREKLQERLAKLAGGVAVIKVGAATETELKEMKLRIEDALNATRAAVEEGIVSGGGTALV
Sau_Gro          DREKLQERLAKLAGGVAVIKVGAASETELKERKLRIEDALNSTRAAVEEGIVAGGGTALV
Bas_Gro          DREKLQERLAKLVGGVAVIKVGAATETELKERKLRIEDALNSTRAAVEEGIVAGGGTSLM
Bsu_Gro          DREKLQERLAKLAGGVAVIKVGAATETELKERKLRIEDALNSTRAAVEEGIVSGGGTALV
                 ************.*******.***:*.*:** * *:***:. :.******:*.***.:*:

Pae_Gro          RALQAIEGLKGDNEEQNVGIALLRRAVESPLRQIVANAGDEPSVVVDKVKQGSGNYGFNA
Msm_Gro          QSAPSLEELSLTGDEAT-GANIVRVALSAPLKQIALNGGLEPGVVAEKVSNLPAGHGLNA
Sag_Gro          NVIEKVAALKLNGDEET-GRNIVLRALEEPVRQIAYNAGYEGSVIIERLKQSEIGTGFNA
Sau_Gro          NVYQKVSEIEAEGDIET-GVNIVLKALTAPVRQIAENAGLEGSVIVERLKNAEPGVGFNA
Bas_Gro          NVYTKVASIVAEGDEAT-GINIVLRALEEPVRQIAINAGLEGSVVVERLKGEKVGVGFNA
Bsu_Gro          NVYNKVAAVEAEGDAQT-GINIVLRALEEPIRQIAHNAGLEGSVIVERLKNEEIGVGFNA
                 .    :  :   .:  . *  ::  *:  *:.**. *.* * .*: :.:.    . *:**

Pae_Gro          ATGVYGDMIEMGILDPAKVTRSALQAAASIGGLMITTEAMVAEIVEDKPAMGGMPDMG--
Msm_Gro          ATGEYEDLLAAGVADPVKVTRSALQNAASIAALFLTTEAVVADKPEKAAAPAGDP-----
Sag_Gro          ANGEWVDMVTTGIIDPVKVTRSALQNAASVASLILTTEAVVANKPEPE--APTAPAMDPS
Sau_Gro          ATNEWVNMLEAGIVDPTKVTRSALQHAASVAAMFLTTEAVVASIPEKNNDQP--------
Bas_Gro          ATGEWVNMLETGIVDPAKVTRSALQNAASVAAMFLTTEAVVADKPEPN--APAMPDMGGM
Bsu_Gro          ATGEWVNMIEKGIVDPTKVTRSALQNAASVAAMFLTTEAVVADKPEENGGGAGMPDMG--
                 *.. : :::  *: **.******** ***:..:::****:**.  *

Pae_Gro          GMGGMGGMM-
Msm_Gro          -TGGMGGMDF
Sag_Gro          MMGGF-----
Sau_Gro          NMGGMPGMM-
Bas_Gro          GMGGMGGMM-
Bsu_Gro          GMGGMGGMM-
                   **:
```

REFERENCES

1. Arora,G. *et al.* Zinc regulates the activity of kinase-phosphatase pair (BasPrkC/BasPrpC) in Bacillus anthracis. *Biometals* **26**, 715-730 (2013).

2. Auger,S., Krin,E., Aymerich,S., & Gohar,M. Autoinducer 2 affects biofilm formation by Bacillus cereus. *Appl. Environ. Microbiol.* **72**, 937-941 (2006).

3. Wessel,D. & Flugge,U.I. A method for the quantitative recovery of protein in dilute solution in the presence of detergents and lipids. *Anal. Biochem.* **138**, 141-143 (1984).

4. Alderwick,L.J. *et al.* Molecular structure of EmbR, a response element of Ser/Thr kinase signaling in Mycobacterium tuberculosis. *Proc. Natl. Acad. Sci. U. S. A* **103**, 2558-2563 (2006).

5. Sajid,A. *et al.* Interaction of Mycobacterium tuberculosis elongation factor Tu with GTP is regulated by phosphorylation. *J. Bacteriol.* **193**, 5347-5358 (2011).

6. Singhal,A. *et al.* Regulation of homocysteine metabolism by Mycobacterium tuberculosis S-adenosylhomocysteine hydrolase. *Sci. Rep.* **3**, 2264 (2013).

7. Schmidl,S.R. *et al.* The phosphoproteome of the minimal bacterium Mycoplasma pneumoniae: analysis of the complete known Ser/Thr kinome suggests the existence of novel kinases. *Mol. Cell Proteomics.* **9**, 1228-1242 (2010).

8. Gupta,M. *et al.* HupB, a Nucleoid-Associated Protein of Mycobacterium tuberculosis, Is Modified by Serine/Threonine Protein Kinases In Vivo. *J. Bacteriol.* **196**, 2646-2657 (2014).

9. Sajid,A. *et al.* Phosphorylation of Mycobacterium tuberculosis Ser/Thr phosphatase by PknA and PknB. *PLoS. One.* **6**, e17871 (2011).

10. Arora,G. *et al.* Unveiling the novel dual specificity protein kinases in Bacillus anthracis: identification of the first prokaryotic dual specificity tyrosine phosphorylation-regulated kinase (DYRK)-like kinase. *J. Biol. Chem.* **287**, 26749-26763 (2012).
