# Supplementary figures and images for: Ser/Thr protein kinase PrkC-mediated regulation of GroEL is critical for biofilm formation in Bacillus anthracis
Source: NPJ Biofilms Microbiomes. 2017 Mar 7;3:7. doi: 10.1038/s41522-017-0015-4 (PMC5460178; doi:10.1038/s41522-017-0015-4)

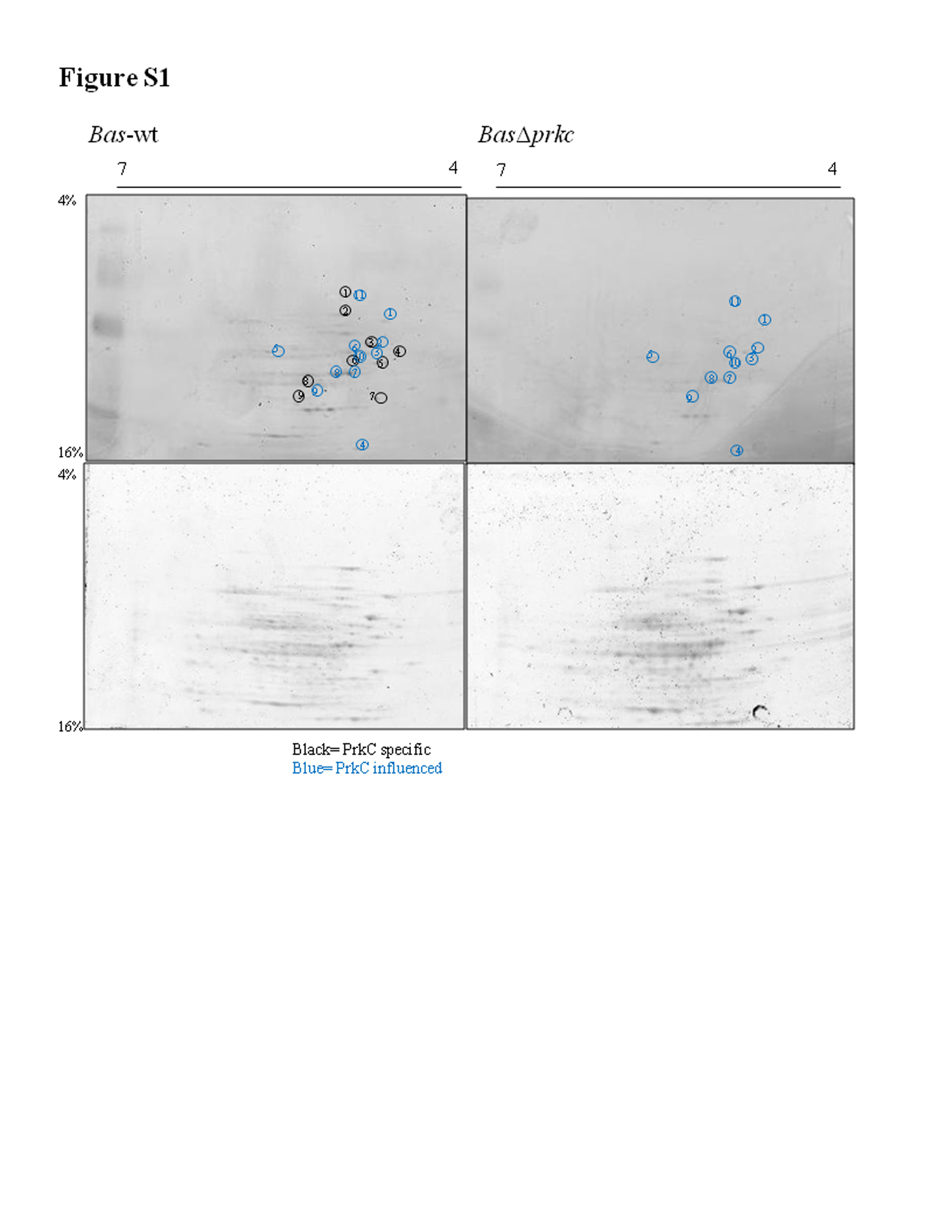

Supplement: Supplementary file 2 — Supplementary Figure 1 [file 41522_2017_15_MOESM2_ESM.tif]

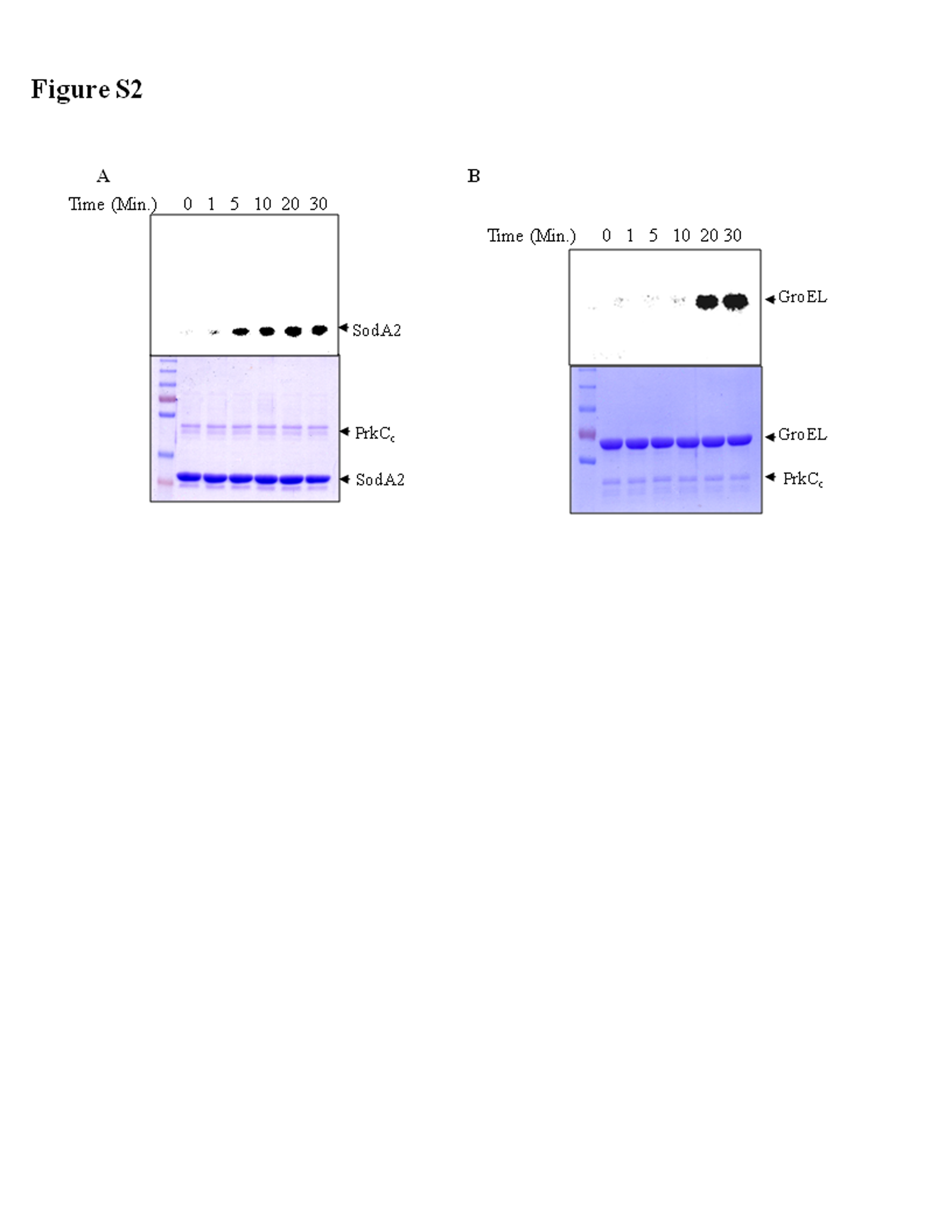

Supplement: Supplementary file 3 — Supplementary Figure 2 [file 41522_2017_15_MOESM3_ESM.tif]

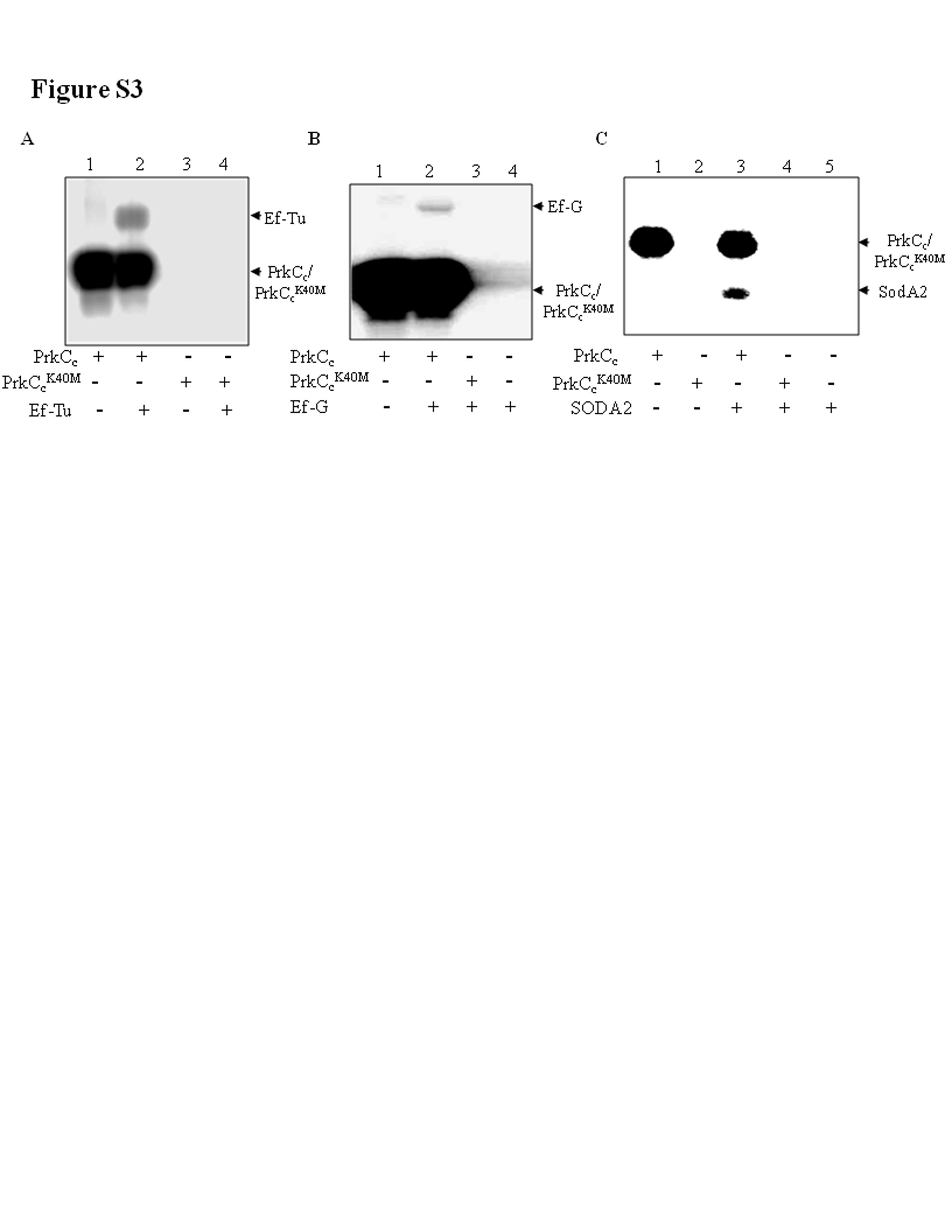

Supplement: Supplementary file 4 — Supplementary Figure 3 [file 41522_2017_15_MOESM4_ESM.tif]
